# Boron-Rich Soft Hydrogels Based on the Coassembly of Cationic A‑B‑A Triblock Copolymers with *Closo*-Dodecaborate

**DOI:** 10.1021/acs.macromol.5c01181

**Published:** 2025-07-10

**Authors:** Soňa Mesíková, Jianwei Li, Sami Kereïche, Zdeněk Tošner, Mariusz Uchman, Miroslav Štěpánek, Michael Gradzielski, Pavel Matějíček

**Affiliations:** 1 Department of Physical and Macromolecular Chemistry, Faculty of Science, 37740 Charles University, Hlavova 2030/8, Prague 2 128 40, Czechia; 2 Institute of Biology and Medical Genetics, First Faculty of Medicine, Charles University and General University Hospital in Prague, Purkynie Ustav, Albertov 4, Prague 12 801, Czechia; 3 NMR Laboratory, Faculty of Science, 37740 Charles University, Hlavova 2030/8, Prague 2 128 40, Czechia; 4 Stranski-Laboratorium für Physikalische Chemie und Theoretische Chemie, Institut für Chemie, Sekr. TC 7, 26524 Technische Universität Berlin, Strasse des 17. Juni 124, Berlin D-10623, Germany

## Abstract

We present an unusual type of electrostatic hydrogel based on anionic boron cluster compounds, which remain almost unexplored in the literature. Cationic PGEA–PEO–PGEA triblock copolymers coassembled with dianionic *closo*-dodecaborate clusters to nanostructures with the PGEA/*closo*-dodecaborate domains with a diameter of around 10–15 nm. Flower-like micelles were observed only at high dilutions, and the dynamic character of the domains was expressed by the formation of bigger particles consisting of multiple domains connected by the PEO bridges. The size of the nanogel particles gradually increased with the sample concentration, leading to macroscopic gelation of the solutions. The SAXS analysis revealed that the shape of the domains is also changing with sample concentration, and isolated short cylinders merge into a continuous phase in the gel-like samples. The rheology measurements showed that the length of the PGEA domain-forming blocks has a significant impact on the mechanical properties of the gels, and the increasing block length leads to stronger and more gel-like systems. All of the gels exhibit marked shear-thinning behavior, which extends over more than five decades of shear rates. Furthermore, the samples are largely self-healing but indicate certain structural reorganization of the network.

## Graphical abstract

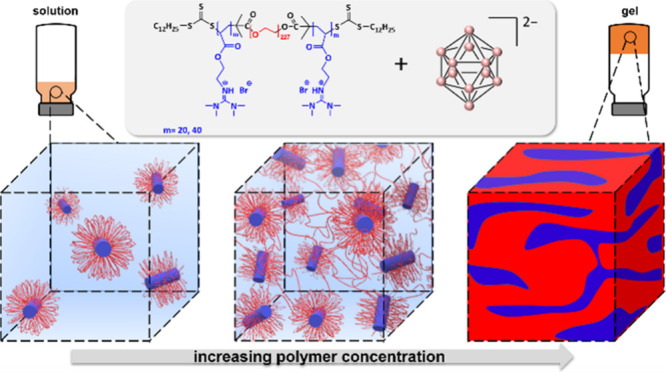

## Introduction

Block copolymers self-assemble to diverse nanostructures in bulk and in selective solvents.^1,2^ The basic concepts to control the nanostructure design and morphology are usually based on precise synthesis of block copolymers of desired absolute and relative block lengths, dispersity, architecture, sequence of blocks, and their interaction parameters,^3–5^ as already recognized in nanochemistry-oriented research because of eminent importance in applications. Further, it was demonstrated that this approach can be successfully extended to the coassembly of hydrophilic homopolymers and double-hydrophilic block copolymers in water.^6–8^

Electrostatic hydrogels are a special class of such nanostructures that can be prepared by coassembly of triblock copolymers (A-B-A), with A being a polyelectrolyte block, with oppositely charged homopolyelectrolytes, diblocks, triblocks, macroions, multivalent ions, etc.^9–14^ Thus, it is based on long-range electrostatic interaction, ensuring their reversibility and tunability.

By introducing multivalent or atypical ions, we can induce coassembly of block copolymers into diverse nanoparticles, nanostructures, and gels driven by a combination of electrostatic and ion-specific interactions, or superchaotropic effect.^15,16^ Anionic boron cluster compounds, ABCCs, and polyoxometalates, POMs, are a unique class of inorganic clusters of nanometer size, which can be considered as building blocks and are perfectly suited for coassembly with polymers.^17–19^

For example, highly symmetrical *closo*-dodecaborates dianions and POM multi-ions are weakly surface active nanoions,^18^ and they induce the formation of nanogel-like particles with cationic block copolymers via simple electrostatic interaction.^8,20^ Besides this, such nanoions are involved in conformational changes of macromolecules in solution, phase transitions, and their adsorption at interfaces, electroneutral surfaces, and molecules.^19^ The term superchaotropicity, coined by Nau and Assaf, is, with a certain precaution, plausible for the description of such solution behavior,^17^ and it was recently put into the context with other nanoions by Horinek and Dullinger.^21^ The superchaotropic effect represents an extreme example of chaotropic behavior, and it has already been recognized as an effect behind the formation of unique nanostructured materials based on electroneutral polymers.^17–19,22,23^ All these nanomaterials have possible applications in drug-delivery, boron neutron capture therapy (BNCT), nanoelectronics, etc.^19^

In this work, the coassembly of cationic triblock copolymers of the A-B-A type with dianionic *closo*-dodecaborate was investigated. Poly­(2-(*N*,*N*,*N*′,*N*′-tetramethyl guanidinium)­ethyl acrylate)-*b*-poly­(ethylene oxide)-*b*-poly­(2-(*N*,*N*,*N*′,*N*′-tetramethyl guanidinium)­ethyl acrylate), PGEA–PEO–PGEA, triblock copolymer in aqueous solutions was used for the formation of hydrogels after mixing with sodium *closo*-dodecaborate, Na_2_[B_12_H_12_]. Polymerization of triblock copolymers was tracked by NMR spectroscopy and SEC. The properties of the gels were studied by diverse scattering, microscopy, and spectroscopy techniques and methods of rheology.

The presented system belongs to a few examples of boron cluster containing polymeric gels in the literature.^20,22,24,25^ The advantage of the system can be seen not only in the presence of boron, which can be utilized in BNCT for the treatment of several types of tumors,^25–27^ but also in its soft and reversible character, which makes it versatile. Last but not least, this is an example of how we can use nanoions with their unique solution properties in producing hybrid nanostructures.

## Experimental Section

### Materials

2-Bromoethanol (95%), acryloyl chloride (97%, contains <400 ppm phenothiazine as stabilizer), triethylamine (TEA, ≥99.5%), 1,1,3,3-tetramethylguanidine (99%), and poly­(ethylene oxide) (PEO) with different molecular weights (around 2, 5, and 10 kDa) were purchased from Sigma-Aldrich. Sodium *closo*-dodecaborate (anhydrous), Na_2_[B_12_H_12_], was purchased from KatChem, Ltd., Czech Republic, and used without further purification. All other reagents were purchased from commercial sources and used as received.

### Synthesis of PGEA–PEO–PGEA Triblock Copolymers

Polymers poly­(2-(*N*,*N*,*N*′,*N*′-tetramethyl guanidinium) ethyl acrylate)-*b*-poly­(ethylene oxide)-*b*-poly­(2-(*N*,*N*,*N*′,*N*′-tetramethyl guanidinium) ethyl acrylate), PGEA_20_–PEO_227_–PGEA_20_ (*M*_n_= 21.7 kg/mol, *Đ* = 1.14), and PGEA_40_–PEO_227_–PGEA_40_ (*M*_n_= 33.5 kg/mol, *Đ* = 1.09), with PEO (*M*_n_ = 10 kg/mol) were synthesized via reversible addition–fragmentation chain-transfer polymerization RAFT; the synthetic route is presented in Scheme 1.

**Scheme 1:**
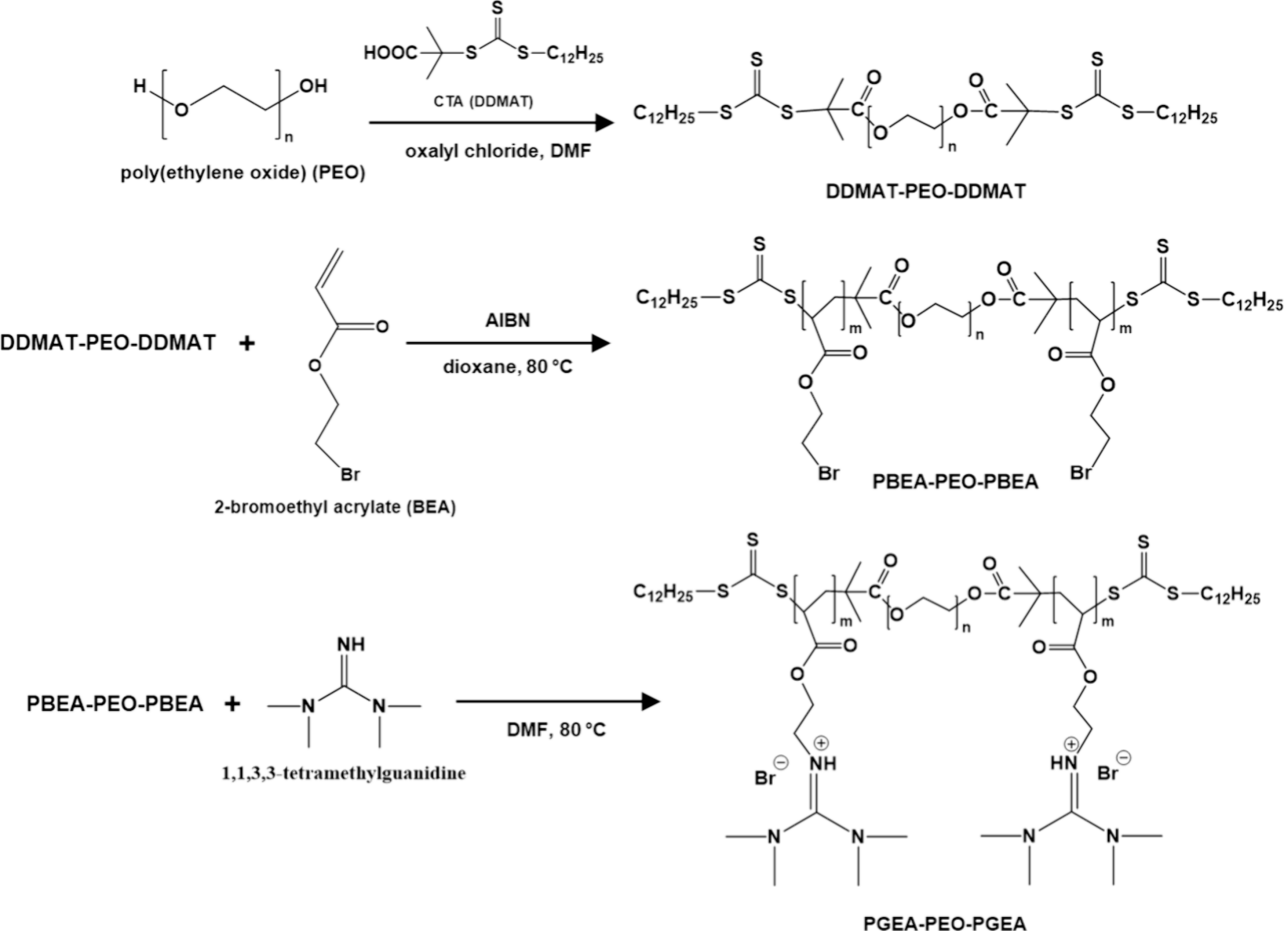
Synthetic Route for Preparation of PGEA–PEO–PGEA Triblock Copolymers; Details Described in the Experimental Section

Initially, 2-(dodecylthiocarbonothioylthio)-2-methylpropanoic acid (DDMAT) was dissolved in dry dichloromethane (DCM), and oxalyl chloride was added slowly to the solution. The reaction mixture was stirred at 30 °C for 2 h, after which the solvent and excess oxalyl chloride were then removed under reduced pressure. The residue was then resolved in dry DCM. Afterward, PEO_227_ in dry DCM was added over 20 min. The reaction mixture was then stirred overnight at 30 °C. The crude product was precipitated in cold diethyl ether and collected by centrifugation. Further purification involved resolving the polymer in THF and precipitating it in cold diethyl ether. Copolymerization with 2-bromoethyl acrylate (BEA) was performed by reversible addition–fragmentation chain-transfer polymerization (RAFT). DDMAT–PEO_227_–DDMAT was dissolved in 1,4-dioxane, in which 2-bromoethyl acrylate was added, followed by three freeze–pump–thaw cycles. Separately, AIBN was dissolved in 1,4-dioxane, performing freeze–pump–thaw cycles three times. After that, the two solutions were mixed together, and the mixture was stirred at 80 °C for 2 h. The resulting copolymer was precipitated in cold diethyl ether. Quaternization of the copolymer by 1,1,3,3-tetramethylguanidine was done by dissolving PBEA-*b*-PEO-*b*-PBEA in dry dimethylformamide (DMF), in which 1,1,3,3-tetramethylguanidine was added. The reaction mixture was stirred overnight at 80 °C. The final product was precipitated in cold diethyl ether and then collected by centrifugation. The series of block copolymers was characterized by standard techniques of size exclusion chromatography (SEC) and NMR spectroscopy (Figures S1a–g and 2a–d in the Supporting Information).

### Sample Preparation

Gels were prepared by mixing triblock copolymers PGEA_20_–PEO_227_–PGEA_20_ and PGEA_40_–PEO_227_–PGEA_40_ (two different PGEA block lengths) with sodium *closo*-dodecaborate (abbreviated as B12) in aqueous solutions at high polymer concentrations (Table 1). The molar ratio of PGEA segments to *closo*-dodecaborate anion was set to a stoichiometric electroneutral value of 0.5. Immediately after the addition of *closo*-dodecaborate, we could observe that the polymer solutions turned into gels (Figure S3 in the SI). The samples were recognized as gel by slow flowing times in the time scales of 1–2 days inspected by a tube inversion experiment. By dilution of the gels, we can lower their viscosity and obtain honey-like systems and even low-viscosity solutions, which probably contain dispersed nanogels and separate flower-like micelles.

**Table 1:** Composition of Samples and Their Visual Description, where *c*(P) is the Concentration of Polymer, *w*(P + B12) is the Weight Fraction of Polymer and *C* *loso*-dodecaborate in Solution, *m*(P) is Weight of the Polymer, and *m*(B_12_) is the Weight of the *Closo*-dodecaborate in Pure Water

PGEA_*m*_	*c*(P)[g/L]	*w*(P + B12) [%]	visual description
20	150	15.0	gel
	147	14.7	gel
	142	14.3	gel
	139	14.1	honey
	135	13.7	high η solution
40	150	15.5	gel
	147	15.3	gel
	142	14.9	gel
	139	14.6	gel
	135	14.3	honey

### Methods

#### Solution-State NMR Spectroscopy

^1^H NMR spectra were measured on a Varian ^UNITY^ *INOVA* 400 in chloroform-*d* and deuterium oxide (99.8%; Chemotrade, Leipzig, Germany). Spectra were referenced to the solvent signal (4.80 ppm).

#### Solid-State NMR Spectroscopy

Solid-state NMR experiments were run on Bruker Avance III HD 400 MHz spectrometer equipped with 3.2 mm CP MAS probe (standard bore) and include (i) one-dimensional single-pulse MAS ^11^B spectrum, ^1^H–^13^C and ^1^H–^11^B CP MAS spectra; (ii) two-dimensional ^1^H–^13^C and ^1^H–^11^B FSLG HETCOR spectra,^28^ with optional ^1^H–^1^H spin-diffusion element (consisting of 90°-delay-90°; zz-mixing) inserted between the *t*_1_ evolution period and the CP block to promote correlations with remote protons. The sample rotation frequency was set to 20 kHz.

#### Size Exclusion Chromatography (SEC)

Size exclusion chromatography (SEC) was performed on an Agilent Technologies 1100 Series apparatus fitted with a UV/vis diode array detector (DAD) and a differential refractometer. Chromatograms from the refractometer are reported. A series of three PL-gel (polystyrene-divinylbenzene) columns (mixed B, mixed C, and mixed E, Polymer Laboratories, UK) and tetrahydrofuran (THF) (flow rate 0.7 mL/min) were used. A set of polystyrene (PS) standards (Polymer Laboratories, UK) was used for column calibration. The following apparent (relative to PS) molecular weight characteristics are reported: number-average molecular weight, *M*_n_, weight-average molecular weight, *M*_w_, and the corresponding averages of degrees of polymerization (DP), *X*_n_ and *X*_w_, respectively, dispersity index *Đ* = *M*_w_/*M*_n_, molecular weight corresponding to the apex of the SEC peak, *M*_p_, and the molecular weight corresponding to the i-th slice of the SEC chromatogram, *M*_i_.

#### Dynamic Light Scattering (DLS)

The dynamic light scattering measurements were performed on a photometer (ALV, Germany) consisting of a CGS-3 automatic goniometer, a 7004 multitau multibit autocorrelator, two high-QE APD pseudo cross-correlation detectors, and a 100 mW, 660 nm diode-pumped solid-state laser (Cobolt AB, Sweden). Dynamic light scattering was performed at a temperature of 23 °C for the scattering angles 50, 90, 130, and 150°. Measurements were carried out for the PGEA–PEO–PGEA concentration region 1–150 g/L with a constant PGEA segment to *closo*-dodecaborate ratio 2:1 in pure water solution, where the measurements were started at the highest concentrations, followed by consecutive dilution by water with a polymer concentration step of 1 g/L. The measured normalized intensity autocorrelation functions were collected and analyzed as described in detail in Results and Discussion. Before measurements and sample preparations, the liquid components were filtered through membrane filters.

#### Small-Angle X-ray Scattering (SAXS)

SAXS experiments were performed with a SAXS point 2.0 (Anton Paar) instrument equipped with a MetalJet C2 (Excillum) X-ray source with lambda = 1.348 Å and with an Eiger R 1 M (Dectris) detector. In the case of liquid samples in the intermediate polymer concentrations (10, 40, and 80 g/L) and stochiometric content of *closo*-dodecaborate dianions in pure water, measurements were conducted at a sample-to-detector distance 0.82 m, corresponding to *q* vectors in the range 0.045–4.1 nm^–1^, in a quartz capillary (1 mm). Gel-like samples with polymer concentration 150 g/L and stochiometric content of *closo*-dodecaborate dianions in pure water were placed in a holder by a tape in a thin layer with a thickness of about 1 mm.

At low dilution, the SAXS data were fitted by the function

$$I\left(q,\eta_{c},\eta_{s},R,\Delta R,L,r,\varphi ,I_{b}\right)=S\left(q,r,\varphi \right)P\left(q,\eta_{c},\eta_{s},R,\Delta R,L\right)+I_{b}$$ 1

where *I*_b_ is background scattering, *S*(*q*, *r*, φ) is the structure factor for hard spheres with the radius *r* and volume fraction φ describing repulsive interactions between block copolymer cylindrical micelles, the scattering of which is treated by the scattering function for the capped cylindrical shell *P*(*q*, η_c_, η_s_, *R*, Δ*R*, *L*), with the scattering length densities (SLDs) of the core η_c_ and of the shell η_s_ (the SLD of the solvent is assumed to be, η_solv_ = 0), the core radius *R*, the shell thickness Δ*R*, and the core length *L*.

The *S*(*q*, *r*, φ) function is expressed as

$$S\left(q,r,\varphi \right)={\left[1+24\varphi \frac{G\left(\varphi ,2qr\right)}{2qr}\right]}^{-1}$$ 2

where

$$G\left(\varphi ,A\right)=\frac{{\left(1+2\varphi \right)}^{2}}{{\left(1-\varphi \right)}^{4}}\left[\frac{\sin A-A\cos A}{A^{2}}\right]-6\varphi \frac{{\left(1+\varphi /2\right)}^{2}}{{\left(1-\varphi \right)}^{4}}\left[\frac{2A\sin A+\left(2-A^{2}\right)\cos A-2}{A^{3}}\right]+\frac{\varphi \alpha }{2}\left[\frac{4\left[\left(3A^{2}-6\right)\cos A+\left(A^{3}-6A\right)\sin A-A^{4}\cos A+6\right]}{A^{5}}\right]$$ 3

The *P*(*q*, η_c_, η_s_, *R*, Δ*R*, *L*) function is expressed as

$$P\left(q,\eta_{c},\eta_{s},R,\Delta R,L\right)=\int_{0}^{1}{\left[K\left(q,\eta_{c}-\eta_{s},R,L,x\right)+K\left(q,\eta_{s},R+\Delta R,L+2\Delta R,x\right)\right]}^{2}dx$$ 4

where

$$K\left(q,\Delta \eta ,R,L,x\right)=2\pi R^{2}L\Delta \eta \frac{J_{1}(qR\sqrt{1-x^{2})}}{qR\sqrt{1-x^{2}}}\frac{\sin\left(qLx/2\right)}{qLx/2}$$ 5

where *J*_1_(*y*) is the first-order Bessel function of the first kind.

SAXS data from gels were fitted by the function composed of a power-law contribution and a broad peak contribution,

$$I\left(q,I_{1},I_{\max},\xi ,m,p,\alpha \right)=I_{1}q^{\alpha }+\frac{I_{\max}}{{\left[1+{\left(|q-q_{\max}|\xi \right)}^{m}\right]}^{p}}+I_{b}$$ 6

where *I*_b_ is background scattering, *I*_1_ is the prefactor for the power-law term with exponent α, *I*_max_ and *q*_max_ are the amplitude and the position of the peak maximum, respectively, ξ is the correlation length, and *m* and *p* are the exponents describing the shape of the peak.

#### Rheology

Rheology measurements were performed using an Anton Paar MCR 501 rheometer with a cone–plate stainless steel geometrical setup. A 25 mm upper rotating cone plate with a cone angle of 1° was used for all measurements with a constant gap size of 0.048 mm. The measurements were conducted at room temperature, 25 °C.

The sample preparation is described in a separate section above. Samples were left to equilibrate for 1 day before the characterization measurements. The volume of samples was 200 μL. The samples were allowed to reach temperature equilibrium for 4 min prior to each measurement.

#### Cryogenic Transmission Electron Microscopy (cryo-TEM)

Cryo-TEM was used to characterize the structure of the nanoparticles in solution. A 3 μL drop of the sample solution was applied to an electron microscopy grid with carbon-covered polymer supporting film (lacey-carbon grids LC200-Cu, Micro to nano, NL), and glow discharged for 30 s with 5 mA current. Most of the sample was removed by blotting (Whatman No. 1 Filter paper) for 1 s, and the grid was immediately plunged into liquid ethane held at −183 °C. The sample was then transferred without rewarming into a JEM 2100Plus (JEOL Ltd., Akishima, Japan) using a Gatan Elsa model 698 cryo-specimen holder (Gatan Inc., Pleasanton, CA). Images were recorded at 200 kV accelerating voltage and microscope magnifications ranging from 15000 to 25000× using a TVIPS TemCam-XF416ES camera (giving a final pixel size from 7.701 to 4.573 Å). The applied underfocus typically ranged between 1.5 and 2.7 μm. The applied blotting conditions resulted in specimen thickness varying between 100 and 300 nm.

#### Isothermal Titration Calorimetry (ITC)

Microcalorimetric titrations were performed in a Nano ITC, TA Instruments (Waters LLC, New Castle, USA). The microcalorimeter consists of a sample cell and a reference cell (24K gold). The sample cell is connected to a 50 μL syringe. The syringe is equipped with a flattened, twisted paddle at the tip, which ensures continuous mixing of the solutions in the cell rotating at 250 rpm. The reaction cell was filled with polymer solutions in water or a 0.1 M NaCl solution (saline). Aliquots of *closo*-dodecaborate solutions in water or saline were subsequently injected into the filled reaction cell. Then, the differential heats were determined for discrete changes of composition. After subtracting the *closo*-dodecaborate dilution heats (obtained from blank experiments), the ITC thermograms were fitted with NanoAnalyze Data Analysis (version 3.10.0, TA Instruments) by a simple one-site binding model to determine the *closo*-dodecaborate/guanidine stoichiometry, *n*, the heat of nanoparticle formation, Δ*H*, the association constant, *K*_a_, and related free energy of the association, Δ*G*, and then the entropy contribution to the free energy of association, −*T*Δ*S.*

## Results and Discussion

### Synthesis of PGEA–PEO–PGEA Triblock Copolymers

For the synthesis of symmetric A-B-A triblock copolymers poly­(2-(*N*,*N*,*N*′,*N*′-tetramethyl guanidinium) ethyl acrylate)-*b*-poly­(ethylene oxide)-*b*-poly­(2-(*N*,*N*,*N*′,*N*′-tetramethyl guanidinium) ethyl acrylate), PGEA–PEO–PGEA, of various block lengths, we employed standard reversible addition–fragmentation chain-transfer polymerization, RAFT, as reported previously for diverse block copolymers.^8,20^ The middle PEO block was used as macroinitiator with the chain-transfer agents on both chain ends, which resulted in a symmetrical A-B-A architecture with two blocks of poly­(2-bromoethyl acrylate), PBEA, on both sides. The PBEA–PEO-PBEA triblock copolymers were subsequently quaternized by tetramethyl guanidinium moieties to the final products, double-hydrophilic polycationic triblock copolymers PGEA_20_–PEO_227_–PGEA_20_ and PGEA_40_–PEO_227_–PGEA_40_ (further details in the Experimental Section and NMR and SEC characterization in the Supporting Information, Figures S1a–g and S2a–d). We can conclude that the synthesized samples are of reasonably low dispersity, however, with evidence of slight tailing caused probably by a small fraction of “dead” chains of PEO in the samples,^29^ which should not have a significant impact on self-assembling and mechanical properties of the system.

### Gelation of PGEA–PEO–PGEA/B12 Mixtures

First of all, to confirm the coassembling behavior of *closo*-dodecaborate dianion (abbreviated as B12 in the further text) with polycationic triblock copolymer PGEA–PEO–PGEA, we conducted the isothermal titration calorimetry (ITC) study at high polymer dilution (concentration around 1 g/L). The results are shown in the Supporting Information (Figure S4 and Table S1), and they are fully in agreement with our previous findings on PEO–PGEA diblock copolymers.^8^ Interaction of B12 with PGEA segments is favorable, with a predominant enthalpy contribution. In addition, the length of the middle PEO block has an impact on the thermodynamic parameters (entropy contribution), which is probably tightly related to the conformation of PEO chains. Given the high dilution, the liquid samples should be composed of free flower-like micelles accompanied by nanogels (a few micelles bridged by PEO blocks). It means that the structure of such nanoparticles can be described as PEO-loops attached to the PGEA/B12 domains, which form compact cores of the nanoparticles. To ensure flexibility of the PEO linkers in the gels, we investigated the samples with the longest PEO blocks in our study (PGEA_20_–PEO_227_–PGEA_20_ and PGEA_40_–PEO_227_–PGEA_40_). As fully hydrogenated *closo*-dodecaborate exhibits only mild superchaotropic behavior in comparison to halogenated clusters,^30^ we assume that electrostatic interaction should be the main driving force for the PGEA/B12 complex formation. Recently, the formation of multiparticle assemblies resembling nanogels was observed in solutions of hydrophilic diblock copolymers with the hydrophobic dodecyl terminal groups originating from the RAFT chain-transfer agent.^31^ Although the PGEA–PEO–PGEA chains are terminated by the same motif, the pure copolymer solutions have low viscosity even at the highest concentrations. Therefore, we do not assume that it contributes significantly to the mechanical properties of the PGEA–PEO–PGEA/B12 gels in comparison to an electrostatic interaction of PGEA segments with B12 dianions.

To investigate the mobility of the PGEA–PEO–PGEA/B12 segments over the broad concentration range in gelly and highly viscous solutions, a simple dynamic light scattering (DLS) experiment was performed. Measurement started with macroscopically gel-like samples at the polymer concentration of 150 g/L, and it was gradually diluted by a step of 1 g/L until a liquid sample with a polymer concentration of 1 g/L was reached. The dilution of the polymers by pure water was controlled by weighing. The most concentrated samples with a gel-like appearance exhibited slow flow in the tube inversion experiment on the time scale of 1–2 days. For the gels, the run time of DLS experiments was set as long as 1 h, which was sufficient for almost all the samples. However, correlation times of the highest concentrations of the PGEA40 sample would require technically unfeasible conditions (measuring on the time scale of days and longer) to get optimal autocorrelation curves. In Figure 1a,b, there are normalized intensity autocorrelation functions, *g* ^(2)^ – 1, presented at an angle of 90°.

**Figure 1:**
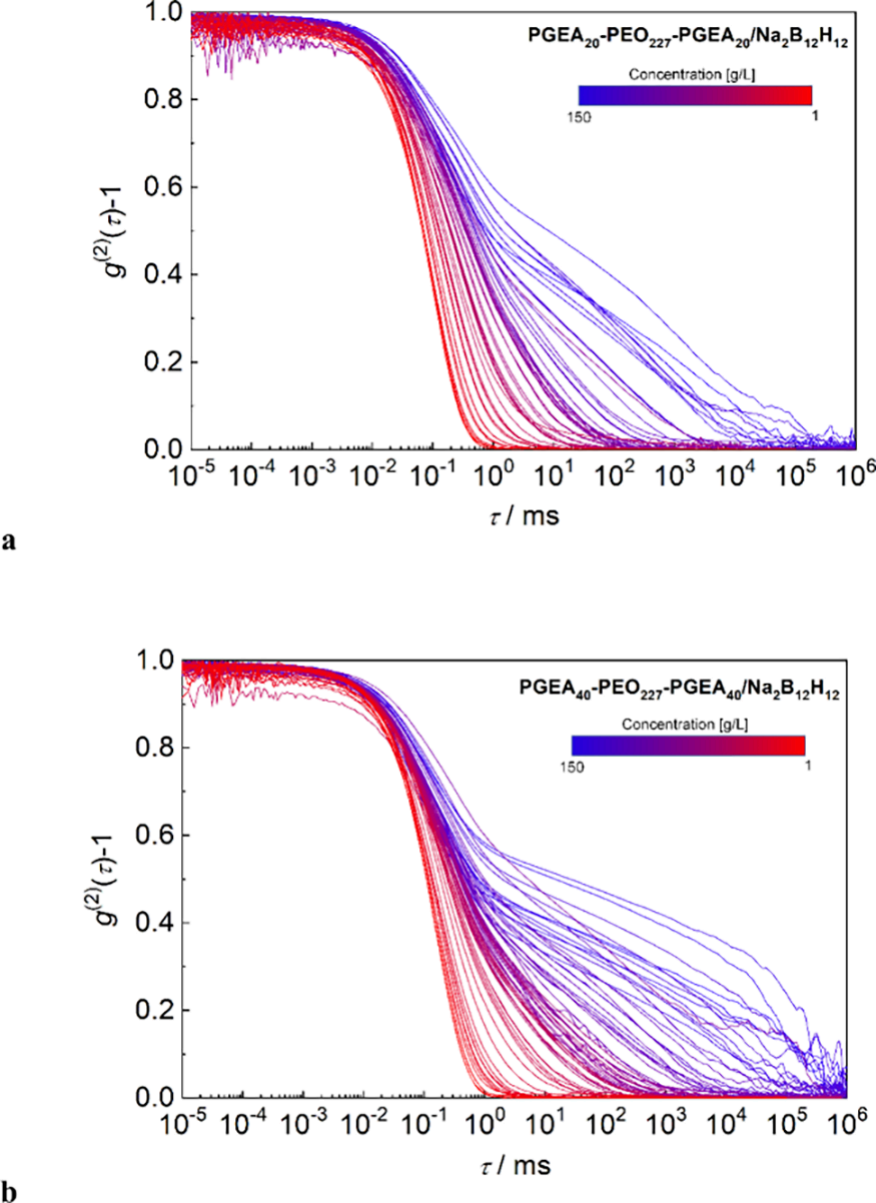
(a, b) Normalized autocorrelation functions of systems. (a) PGEA_20_–PEO–PGEA_20_/B12 and (b) PGEA_40_–PEO–PGEA_40_/B12 at the range of concentrations from 150 to 1 g/L.

For the broad range of polymer concentrations, the coexistence of a fast mode and a distinct slow mode is clearly observed in both samples. Both modes are diffusive (shown in Figure S5 and SI). The fast mode is most likely related to the free diffusion of flower micelles, while the slow mode is related to the collective motion of larger gel particles. Note that the presence of the slow mode is evident even in the samples that are visually liquid (Table 1). We fitted all the normalized autocorrelation functions shown in Figure 1a,b by a combination of exponential and stretched exponential functions as follows:

$${\left[g^{\left(2\right)}\left(t\right)-1\right]}^{1/2}=A_{1}\exp\left[-\frac{t}{\tau_{1}}\right]+A_{2}\exp\left[-{\left(\frac{t}{\tau_{2}}\right)}^{p}\right]$$ 7

where *A*_1_ and *A*_2_ are the amplitudes of the fast and slow modes; τ_1_ and τ_2_ are the correlation times of the fast and slow modes, respectively; and *p* is the stretched exponent of the slow mode.^14,32^ The results are shown in Figures S6 and S7 in the SI, where the values of *A*_1_, *A*_2_, τ_1_, τ_2_, and *p* are plotted against the polymer concentration.

We can see that the slow mode is prevalent (Figure S7a) in the samples with a polymer concentration exceeding 50 g/L. The correlation times (Figure S7b) show the same trends for both samples: The values of τ_1_ are practically constant in the whole concentration range (time scale ca. 10^–1^ ms). For both samples, the hydrodynamic radii, *R*_h_, of free-moving micelles were calculated from the corresponding correlation times, τ_1_, shown in Figure S7b at high dilution (below 50 g/L), using relations 1/τ_1_ = *Dq* ^2^ and *R*_h_ = *k*_B_ *T*/6πη*D*, where *D* is diffusion coefficient, *q* magnitude of scattering vector, *k*_B_ Boltzmann constant, *T* temperature, and η solvent viscosity, to be around 15 and 19 nm for PGEA_20_–PEO–PGEA_20_/B12 and PGEA_40_–PEO–PGEA_40_/B12 micelles, respectively. In contrast, the correlation times of the slow mode vary significantly with polymer concentration. However, the system exhibits a gradual decrease rather than a distinct break with sample dilution. The visually gel-like samples have the values of τ_2_ in the time scales exceeding 10^3^ ms, but they level off at the time scales ca. 10^0^ ms below concentrations of 50 g/L. We do not see any distinct break-even in the dependence of the stretched exponent *p* (Figure S7c). Its values increase from 0.15 to ca. 0.6 upon dilution for both samples. We can interpret it that the systems at the highest concentrations are close to the power-law behavior of viscoelastic materials, where *p* = 0, and the corresponding critical exponents are related to the fractal dimension of branching in nonergodic gels.^32^ The value of *p* gradually increases with dilution, and then the slow mode probably corresponds to the collective diffusion of nano- or microgel particles.

### Morphology of PGEA/B12 Domains in Micelles and Gels

The morphology of PGEA–PEO–PGEA/B12 was investigated by the SAXS technique over the broad concentration range. Prior to the scattering study, we visualized very dilute liquid samples by the cryo-TEM technique. The concentrated and high-viscosity samples were not visualized, as they are tricky to treat by cryo-TEM because of the sample handling and a particle “overcrowding”. The corresponding micrographs are shown in Figure 2. As is typical for the soft core–shell particles, the POE shell has too low contrast to be visualized, and dark stains in the micrographs can be assigned to the PGEA/B12 domains. The size distributions are presented in histograms in Figure 2. It is evident that PGEA20/B12 domains form small, roughly spherical objects with diameters around 11 nm. In comparison, the PGEA40/B12 domains are slightly bigger with a diameter around 14 nm, nevertheless with partly distorted sphericity and with increased dispersity. However, these numbers are in accordance with hydrodynamic diameters of around 30 nm determined for the core–shell micelles by DLS.

**Figure 2:**
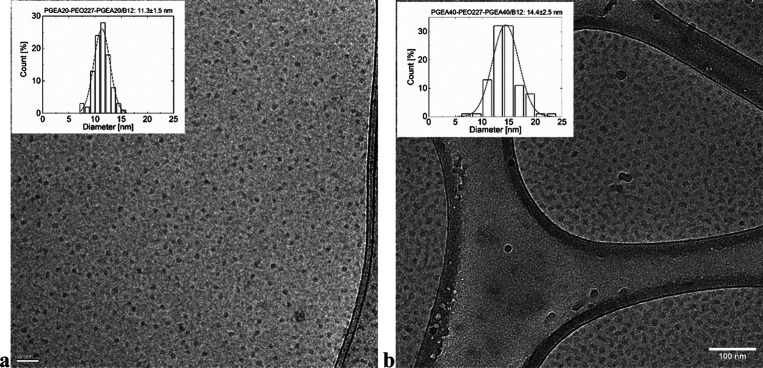
(a, b) Cryo-TEM micrographs of very dilute liquid samples of (a) PGEA_20_–PEO–PGEA_20_/B12 and (b) PGEA_40_–PEO–PGEA_40_/B12 at the polymer concentration of 1 g/L.

As already outlined, liquid samples at elevated polymer concentrations (10, 40, and 80 g/L) and gel-like samples (150 g/L) were investigated by SAXS. The scattering curves fitted by physically plausible models based on the nanoparticle visualization by cryo-TEM are shown in Figure 3. While the liquid samples were measured in capillaries and the scattering curves could be recalculated to the absolute scale, this was not the case in the gel-like samples, which were measured in layers fixed by a tape. Such scattering curves are plotted on an arbitrary scale only.

**Figure 3:**
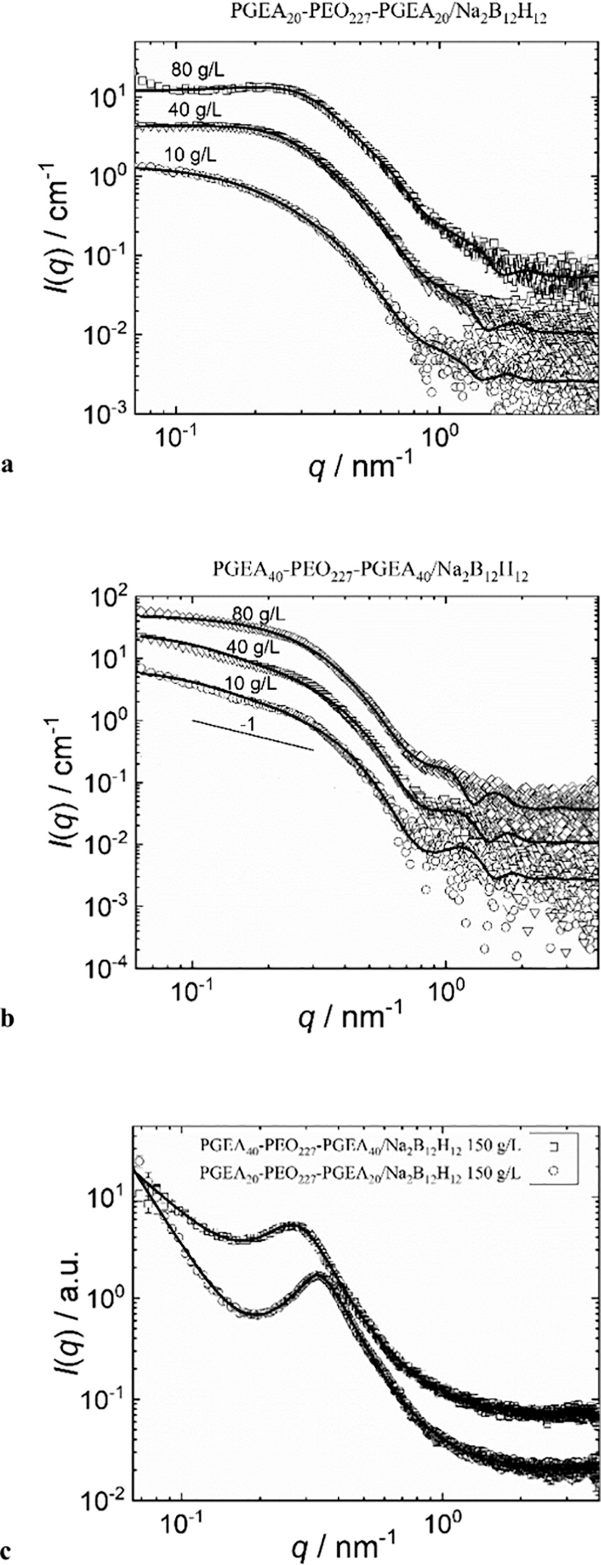
(a–c) SAXS curves with corresponding fits of liquid samples of (a) PGEA_20_–PEO–PGEA_20_/B12 and (b) PGEA_40_–PEO–PGEA_40_/B12 in the concentration range 10–80 g/L, and (c) gel-like samples at the concentration 150 g/L.

The liquid samples were successfully modeled as a system of short core–shell cylinders (model described in Experimental and the results of modeling in Table S2, Supporting Information). The length-to-diameter ratios with both core and shell included are around 1 and 3 for PGEA20 and PGEA40 samples, respectively. In line with the morphology visualized by cryo-TEM at 1 g/L, the nanoparticles only slightly deviate from the spherical shape, which is pronounced especially for the PGEA40 system. We could see mild changes in scattering curves and the parameters of the fit with increasing concentration, which indicates certain changes in morphology. In the case of the PGEA20 sample, the structure factor had to be included, indicating partial particle crowding.

The scattering curves of the gels are significantly different in comparison to those of the liquid samples. Most likely, soft nanoparticles can change their shape and morphology with increasing polymer concentration. The scattering curves of gel could not be modeled by a system of separate core–shell particles anymore, but they were fitted as bicontinuous phases by a model combining a power-law contribution and a broad peak contribution (model described in Experimental Section and the results of modeling in Table S3, Supporting Information). The PGEA20 and PGEA40 systems differ in the position of the correlation peak (related to the domain packing) and the value of the correlation length (related to the mesh size of the network). So, the center-to-center distances and the mesh sizes are 19 and 9 nm for the PGEA20 sample and 23 and 11 nm for the PGEA40 sample, respectively. Further, the fractal dimensions of the networks are 4.1 and 2.4 for the PGEA20 and PGEA40 samples, respectively. We assume that the core–shell morphology is retained even after the merging of flower micelles or small micellar aggregates into the macroscopic gel. Then, we can assign the above-mentioned bicontinuous phases to the merged PGEA/B12 domains and to the PEO chains extensively swelled by water.

The electrostatic character of PGEA/B12 complexation was tested by SAXS scattering experiments with the PGEA–PEO–PGEA/B12 nanoparticles in 0.1 M NaCl solutions (Figure S8 and Table S4 in the SI).

In conclusion, the morphology of the PGEA20 gel is, on average, more closely packed and less fuzzy than that of the PGEA40 sample. However, both systems seem to be fully amorphous without any significant long-range ordering. The representative scheme of the flower micelle to nanogel to bicontinuous gel transition is shown in Figure 4. The sharp transition of macroscopically fluid solutions to gels after crossing the gel point is well-known in the literature and can be described by the percolation model.^33^ In our case, the percolation point should be higher than the overlap concentration of free expanded polymer chains because the macroscopic gel is formed by random merging of compact PGEA/B12 domains, decorated and at the same time interconnected by water-swollen PEO chains.

**Figure 4:**
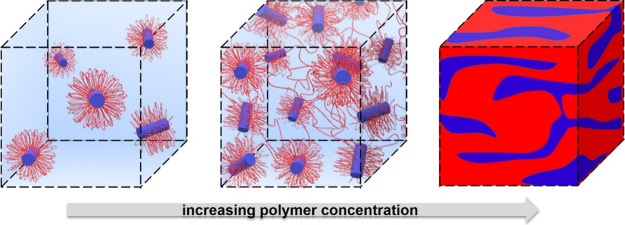
Schematic picture of PGEA–PEO–PGEA/B12 nanostructures at various concentration. While flower-like micelles and nanogels with the shape of short cylinders are present in liquid samples at low concentrations, bicontinuous phase with merged PGEA/B12 domains are present in macroscopically gel-like systems. Color coding: water (light blue), PEO shell (red lines), PGEA/B12 domains (blue bodies and blue area), and PEO phase swelled by water (red area).

### Structural Motif of Tetramethyl Guanidinium/*Closo*-dodecaborate

To better understand the driving forces behind the coassembly of *closo*-dodecaborate with tetramethyl guanidinium-based A-B-A triblock polycations leading to cylindrical nanostructures, we attempted to reveal the packing and mutual interactions between cationic and anionic moieties within the soft hydrogel environment. As the system is too complex, we prepared a simpler solid system that is more suitable for the structural analysis: the precipitant of homopolymer poly­(2-(*N*,*N*,*N*′,*N*′-tetramethyl guanidinium)­ethyl acrylate), PGEA_40_, (SEC traces and NMR spectrum shown in Figures S1f,g and S2d in the SI) with *closo*-dodecaborate. The precipitant of PGEA_40_/B12 was studied by means of solid-state NMR spectroscopy. We performed ^1^H–^13^C and ^1^H–^11^B FSLG heterocorrelation (HETCOR) NMR experiments (Figures S9 and S10 in the SI).^28,34^ The spectra revealed the spatial proximity of *closo*-dodecaborate clusters and methyl groups attached to the N atoms of guanidinium.

We hypothesize that the complex formation is most likely driven by simple electrostatic attraction, and it is not stabilized by weak interactions such as dihydrogen bonding, which was observed between B–H and N–H units in the guanidinium/B12 crystal structure.^35^ The methylation of the cationic moieties in PGEA probably prevents the formation of such bonds in our samples.

The reason why PGEA/B12-based nanostructures prefer worm-like and cylindrical morphologies is still not clear, but it is probably given by the flat triangular shape and potential stacking of tetramethylguanidinium moieties. The packing into cylindrical nanostructures was recently also described for poly­(vinylpyridinium)/COSAN nanocomposites, and it was probably caused by the bulkiness of boron cluster nanoions.^34^

### Rheological Properties of PGEA–PEO–PGEA/B12 Gels

Of course, for gel-like or highly viscous samples, their rheological properties are of central importance for their understanding. Accordingly, these systems were studied by oscillatory and shear rheology. Here, one may keep in mind that these techniques address the relaxation processes in the systems, and these observations are related to the relaxations seen in DLS, but in rheology, one probes macroscopic relaxation and for longer time scales. However, the relation between macroscopic and microscopic relaxations has been found and described in the literature.^36^

For both PGEA–PEO–PGEA copolymers (the PGEA block lengths 20 and 40), samples of polymer concentration 150, 147, 142, 139, and 135 g/L in pure water and in 0.1 M NaCl solution were prepared, in order to also address the relevance of electrostatic interactions for the viscoelastic properties. The PGEA segment to *closo*-dodecaborate molar ratio was set to the stoichiometric value of 0.5 (Table 1). Samples were left to equilibrate for 1 day before the measurement. The volume of samples was 200 μL. For all cases, the rheology data indicate that the gels prepared in 0.1 M NaCl solutions are softer and they more easily liquify upon dilution in comparison to the systems in pure water (data shown and further discussed in the SI). This is another indication that the PGEA-B12 interaction is electrostatically driven.

Looking at the rheology of the samples prepared with pure water, one finds from an amplitude sweep (Figure S11) that all samples can sustain a deformation of 10% without any effect on their viscoelastic moduli. Oscillatory rheology experiments were done in the linear viscoelastic regime in the frequency range of 0.1 to 100 rad/s (Figure S12) and show that for both PGEA20 and PGEA40 samples, in the concentration range 142–150 g/L, the values of storage modulus *G*′ were higher than those of loss modulus *G*″ for higher frequencies, confirming their gel character. In addition, the moduli show only a rather small frequency dependence, in general continuously increasing with increasing frequency. For the PGEA20 system, it is interesting to note that at low frequencies, *G*″ becomes larger than *G*′ (best seen in Figure S13, which shows tanδ = *G*″/*G*′). From the crossover point, one can conclude to a structural relaxation time of these gels of about 1–2 s. For the lowest concentration of 139 g/L, the viscoelastic moduli are almost 3 orders of magnitude less, showing the much more liquid-like character, and here, *G*″ is generally higher than *G*′. For the PGEA40 system, the moduli are generally somewhat higher, especially for the most dilute samples, and show less of a frequency dependence. Also, they show no crossover for *G*′ and *G*″ (see tanδ in Figure S13), which means that their structural relaxation time must be well above 10 s. In general, these experiments confirm the more pronounced gel character of the PGEA40 samples compared to that of the equivalent PGEA20 samples.

The frequency sweep (Figure S12) was carried out first with increasing radial frequency (1st step), followed by measuring the same points at decreasing radial frequency (2nd step). In general, we observed only a small to negligible hysteresis of *G*′ and *G*″. Only below 1 rad/s can one observe somewhat different values, and this effect is more significant for the PGEA20 sample. It could be caused by sample slipping or by changes in electrostatic interactions within the PGEA/B12 domains.

As already mentioned in the discussion of the DLS experiments, the gel-like samples are close to the power-law behavior of viscoelastic solids. According to the literature, the frequency sweep data were fitted by a function *G*′ = *A* + *B*ω^*C*^, where *A*, *B*, and *C* are empirical parameters.^37–39^ The values of the power-law parameter *C* are listed in Tables S5 and S6 in the SI, and they indicate that the PGEA40 sample in pure water forms elastic gels over all the measured concentrations (*C* close to 0). The PGEA20 samples also form rather elastic gels at high polymer concentrations, but they rapidly turn to viscous gels with the sample dilution (*C* close to 1). Similar behavior was observed for the PGEA20 samples in 0.1 M NaCl, but the transition to viscous gel occurred at higher polymer concentration. It is even pronounced for the PGEA40 samples in salt solutions, where the value of *C* is as high as 2 at 139 g/L, which corresponds to dilute aqueous solutions.

For a better comparison of the different systems, the storage moduli *G*′ at radial frequency 10 rad/s are plotted in Figure 5 and Figure S14. We can see that a decrease of *G*′ with decreasing concentration occurs below 145 g/L for both systems, but is much more pronounced for the PGEA20 system. Apparently, the length of the PGEA block has an impact on the strength of interactions within the PGEA/B12 domains.

**Figure 5:**
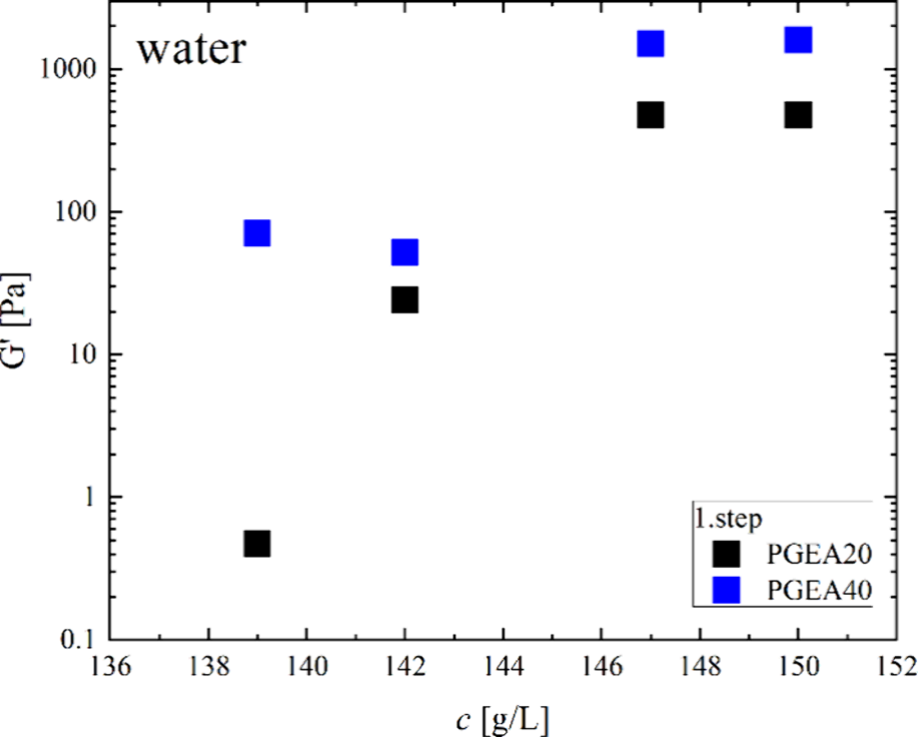
Storage modulus *G*′ for the PGEA20 and PGEA40 samples in pure water for different concentrations at radial frequency 10 rad/s and *T* = 25 °C; 1. step, where frequency sweep is measured from low to high frequencies, is shown; 2. step, where the experiment is performed from high to low frequencies, is shown in Figure S14 in the SI.

Interestingly, tan­(δ) (= *G*″/*G*′), which is a measure for the relative strengths of viscous and elastic components, hardly changes with concentration, except for the most dilute PGEA20 sample, but here also, the measured values were so low that they were not so reliable any more (Figure S13). For the PGEA40 samples, one observes for frequencies above 1 rad/s an increase in the relative elastic properties with increasing concentration.

In the next step, constant shear experiments were done in which shear rates γ̇ of 0.01 to 1000 1/s were applied. For all samples marked shear-thinning is observed, except for the most dilute PGEA20 sample, where the viscosity from the beginning is not so high (∼1 Pa s) and the observed shear thinning is accordingly less pronounced (Figure 6). For the other samples, viscosities at the lowest shear rate in the range of 10^3^–10^5^ Pa s are seen, with generally higher values for the PGEA40 systems. Here, the shear-thinning following a power law, γ̇^–1^ is seen, while it is taking place less sharply for the PGEA20 systems. Also certainly interesting is that for the most viscous systems, the shear-thinning takes place over more than five decades of shear rates, which indicates a marked power-law response of the viscoelasticity (Figure 6). In contrast, the PGEA20 samples display plateaus at low and high shear rates, thereby indicating also that they are not really gel-like but have characteristic structural relaxation times in the range of 5–50 s, in agreement with the observations in the oscillatory experiments (Figure 4). At the moment, we have no explanation for the steps in the shear deformation experiments observed, especially for PGEA20 samples at the highest concentrations.

**Figure 6:**
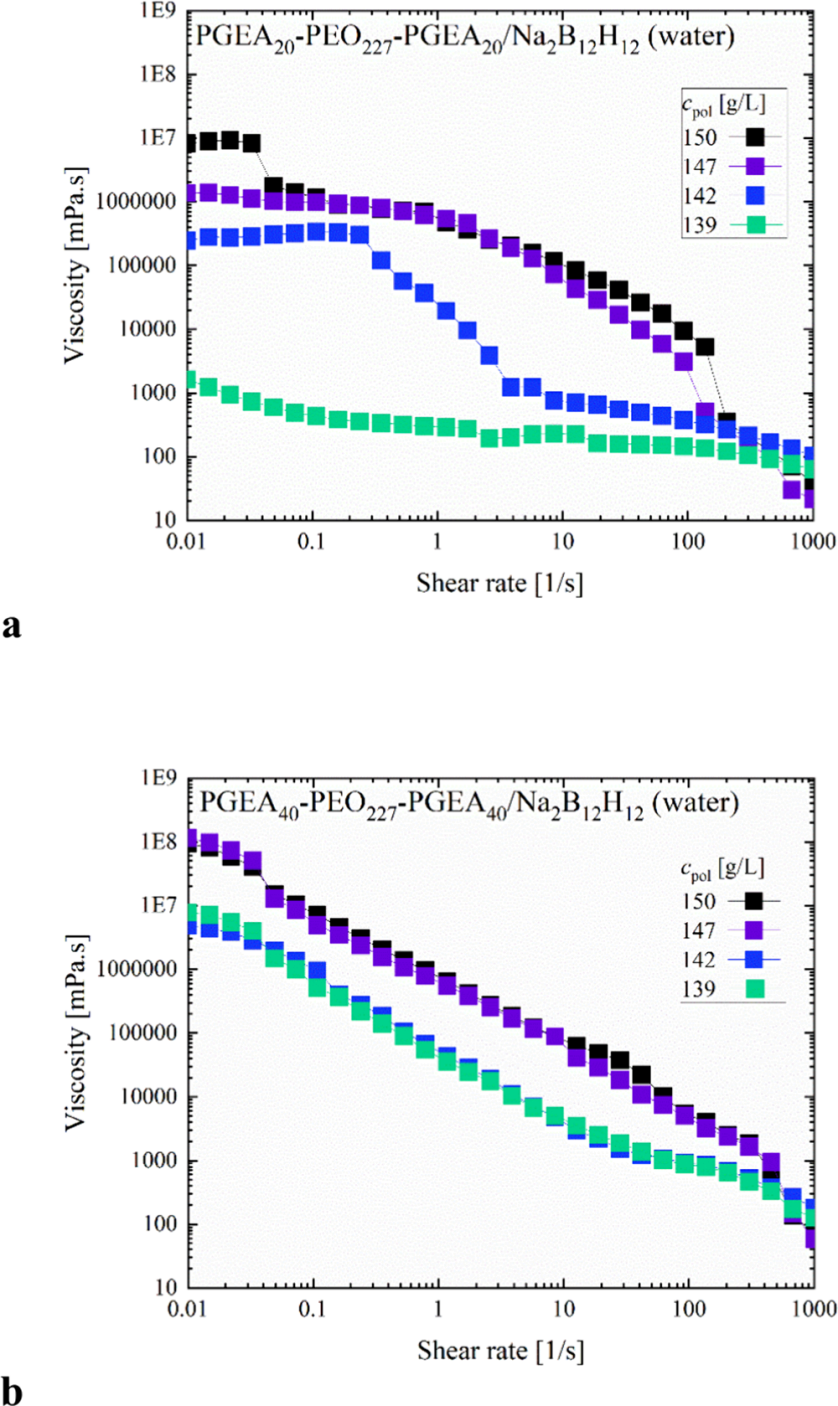
(a, b) Shear deformation experiment for (a) PGEA20 and (b) PGEA40 samples at concentrations 150, 147, 142, 139, and 135 g/L in pure water (*T* = 25 °C).

After the cessation of shear in the constant shear experiments, which definitely disrupted the initially present network structures, a self-healing test was carried out in order to see if and how rapidly the initially present network structure is recovered (Figure 7). For this purpose, the viscoelastic moduli were recorded for a period of 30 min at a radial frequency of 10 rad/s. For all the samples, we observe a substantial recovery. The values of *G*′ and *G*″ grow during the self-healing test, which lasted 30 min. However, this process is rather slow, and for all samples, one still sees substantial changes in the moduli even after 30 min. For the PGEA40 sample, at concentrations 150 and 147 g/L, *G*′ and *G*″ do not reach the values from the frequency sweep in 30 min. However, for the concentrations 142 and 139 g/L, *G*′ and *G*″ reach the values from the frequency sweep. For PGEA20 samples, *G*′ reaches the values from the frequency sweep at concentrations 150, 147 g/L, but *G*″ does not. At concentrations 142 and 139 g/L, both *G′* and *G″* reach the frequency sweep values within 30 min. In many cases, *G′* not only reaches but exceeds the frequency sweep values at 10 rad/s. One likely explanation for this could be better rearrangement of the gel network after deformation.

**Figure 7:**
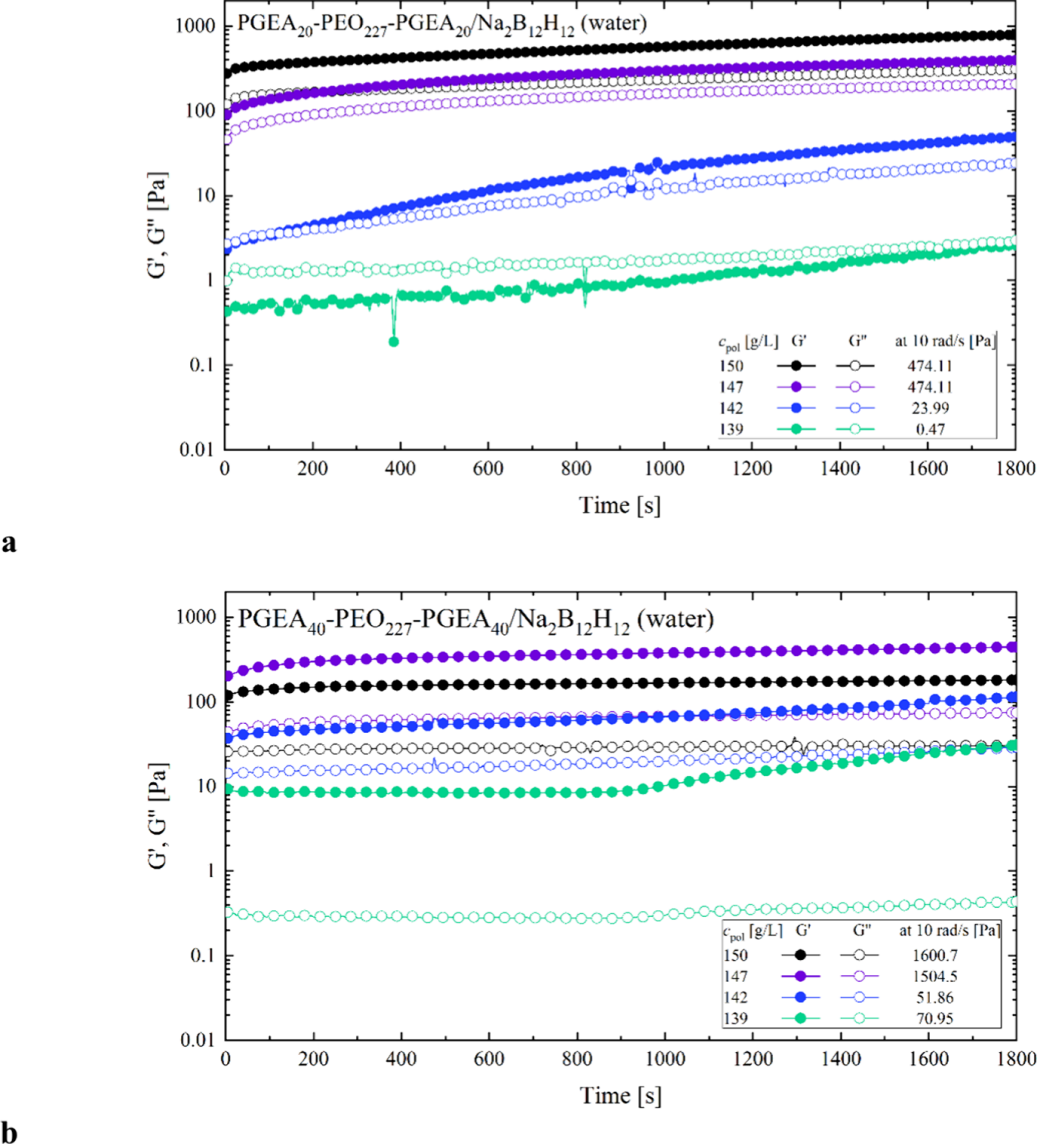
(a, b) Self-healing test for (a) PGEA20 and (b) PGEA40 samples at concentrations 150, 147, 142, 139, and 135 g/L in pure water, where storage modulus is represented by full lines and loss modulus is represented by dotted lines (*T* = 25 °C); the values of storage modulus obtained by oscillating at radial frequency of 10 rad/s are listed in the inset.

The same experiments were also performed with the same samples in 0.1 M NaCl instead of pure water. The obtained curves for the amplitude sweeps (Figure S15) and the frequency sweeps (Figure S16) look similar to those for the case in pure water, but the observed moduli are generally lower than for the equivalent systems in pure water. The concentration dependence is more marked than for the pure water systems (Figure S17) and generally, the softening of the gels occurs at higher concentration. The relative elasticity of the systems, quantified by tanδ, is generally lower than that for the pure water systems (Figure S18). As the viscosities are generally somewhat lower than for the pure water systems, the effect of shear-thinning is less marked. Only in the most concentrated PGEA40 systems does it still occur over the whole shear rate range probed, but initially with a much lower power law. The reason for the sudden increase of the power law above 50 1/s is unclear. For the self-healing test, one has the interesting observation that it takes place faster than in pure water. This demonstrates that the required reorganization of the network structure becomes facilitated by the reduced electrostatic interaction.

In conclusion, the rheology measurements showed that the length of the PGEA/B12 domain-forming blocks has a significant impact on the mechanical properties of the gels. The PGEA40 gels with longer PGEA blocks are stronger and much more gel-like than the PGEA20 systems. All the gels exhibit marked shear-thinning behavior, especially the PGEA40 samples, which here extends over more than five decades of shear rates. Furthermore, the samples are largely self-healing, but this process takes rather long, thereby indicating that the required structural rearrangement requires substantial reorganization of the network. In salt solution (0.1 M NaCl), the gels are weaker, but they exhibit better self-healing properties in comparison with the same systems in pure water because of the screening of electrostatic attraction in the PGEA/B12 domains.

## Conclusions

In this work, the coassembly of cationic triblock copolymers of the A-B-A type with atypical boron cluster nanoions was investigated. PGEA–PEO–PGEA triblock copolymer interacts with sodium *closo*-dodecaborate, leading to the PGEA/*closo*-dodecaborate domains with a diameter around 10–15 nm. Simple flower-like micelles were observed by dynamic light scattering and cryo-TEM imaging only at high sample dilutions. The highly dynamic character of the domains leads to the formation of bigger particles consisting of multiple domains interconnected by the PEO blocks at higher sample concentrations. The size of such nanogel particles is gradually increasing with the sample concentration, leading to macroscopic gelation at a polymer concentration of above 130 g/L. The SAXS analysis revealed that the shape of the domains is also changing with sample concentration, and isolated short cylinders were observed only in the liquid-like samples. It seems that the macroscopically gel-like samples consist of bicontinuous phases where the PGEA/*closo*-dodecaborate domains are interconnected. We hypothesize that the PGEA/*closo*-dodecaborate formation is most likely driven by simple electrostatic attraction, and it is not stabilized by weak interactions such as dihydrogen bonding because of the methylation of the cationic moieties in PGEA segments.

The rheology measurements showed that the length of the PGEA blocks has a significant impact on the mechanical properties of the gels. The gels with longer PGEA blocks are stronger and much more gel-like. All of the gels exhibit shear-thinning behavior. Furthermore, the samples are largely self-healing, but this process takes rather long, thereby indicating that the required structural rearrangement requires substantial reorganization of the network. The addition of NaCl to the samples leads to weaker gels but with better self-healing properties, which is related to the screening of electrostatic attraction within the PGEA/*closo*-dodecaborate domains.

## Supplementary Material



## Notes

Polymer synthesis; NMR and SEC characterization of polymers; appearance of gel-like samples; characterization of the samples by isothermal titration calorimetry, dynamic light scattering, small-angle X-ray scattering, solid-state NMR spectroscopy; mechanical properties and additional rheology analysis of the samples (PDF)

## References

[1] Mai Y., Eisenberg A. (2012). Self-assembly
of block copolymers. Chem. Soc. Rev..

[2] Blanazs A., Armes S. P., Ryan A. J. (2009). Self-Assembled
Block Copolymer Aggregates:
From Micelles to Vesicles and their Biological Applications. Macromol. Rapid Commun..

[3] Riess G. (2003). Micellization
of block copolymers. Prog. Polym. Sci..

[4] Zhang L., Eisenberg A. (1995). Multiple Morphologies
of ″Crew-Cut″ Aggregates
of Polystyrene-b-poly­(acrylic acid) Block Copolymers. Science.

[5] Foerster S., Khandpur A. K., Zhao J., Bates F. S., Hamley I. W., Ryan A. J., Bras W. (1994). Complex Phase Behavior
of Polyisoprene-Polystyrene
Diblock Copolymers Near the Order-Disorder Transition. Macromolecules.

[6] Voets I. K., de Keizer A., Cohen Stuart M. A. (2009). Complex coacervate core micelles. Adv. Colloid Interface Sci..

[7] Uchman M., Pispas S., Kovacik L., Stepanek M. (2014). Morphologically
Tunable
Coassembly of Double Hydrophilic Block Polyelectrolyte with Oppositely
Charged Fluorosurfactant. Macromolecules.

[8] Li J., Janouskova O., Fernandez-Alvarez R., Mesikova S., Tosner Z., Kereiche S., Uchman M., Matejicek P. (2020). Designed Boron-Rich
Polymeric Nanoparticles Based on Nano-ion Pairing for Boron Delivery. Chem. - Eur. J..

[9] Senebandith H., Li D., Srivastava S. (2023). Advances, Applications, and Emerging Opportunities
in Electrostatic Hydrogels. Langmuir.

[10] Stano R., Kosovan P., Tagliabue A., Holm Ch. (2021). Electrostatically
Cross-Linked Reversible Gels-Effects of pH and Ionic Strength. Macromolecules.

[11] Huang X., Zhang A., Tan Q., Gou K., Chen Y., Nie Y., Weng G. (2024). Highly Stretchable
and Self-Healable Tough Polymer
Elastomer through Dy3+ and Cu2+ Coordination Cross-Linking. Macromolecules.

[12] Nakagawa Y., Oki Y., Da X., Singh Chandel A. K., Ohta S., Ito T. (2022). Injectable
bottlebrush triblock copolymer hydrogel crosslinked with ferric ions. Polymer.

[13] Laquerbe S., Es Sayed J., Lorthioir C., Meyer C., Narita T., Ducouret G., Perrin P., Sanson N. (2023). Supramolecular Crosslinked
Hydrogels: Similarities and Differences with Chemically Crosslinked
Hydrogels. Macromolecules.

[14] Ozaki H., Indei T., Koga T., Narita T. (2017). Physical gelation
of
supramolecular hydrogels cross-linked by metal-ligand interactions:
Dynamic light scattering and microrheological studies. Polymer.

[15] He Q., Zhang Y., Li H., Chen Q. (2020). Rheological Properties
of ABA-Type Copolymers Physically End-Cross-Linked by Polyoxometalate. Macromolecules.

[16] Wei H., Du S., Liu Y., Zhao H., Chen C., Li Z., Lin J., Zhang Y., Zhang J., Wan X. (2014). Tunable, luminescent,
and self-healing hybrid hydrogels of polyoxometalates and triblock
copolymers based on electrostatic assembly. Chem. Commun..

[17] Assaf K. I., Nau W. M. (2018). The Chaotropic Effect
as an Assembly Motif in Chemistry. Angew. Chem.,
Int. Ed..

[18] Matejicek P. (2020). Erratic ions:
self-assembly and coassembly of ions of nanometer size and of irregular
structure. Curr. Opin. Colloid Interface Sci..

[19] Li J., Kim J. S., Fan J., Peng X., Matejicek P. (2025). Boron cluster
leveraged polymeric building blocks. Chem. Soc.
Rev..

[20] Li J., Fernandez-Alvarez R., Tosner Z., Kereıche S., Uchman M., Matejicek P. (2021). Engineered
nanogels shape templated
by closo-dodecaborate nano-ion and dictated by chemical crosslinking
for efficient boron delivery. J. Mol. Liq..

[21] Dullinger P., Horinek D. (2023). Solvation of Nanoions
in Aqueous Solutions. J. Am. Chem. Soc..

[22] Hohenschutz M., Bauduin P., Lopez C. G., Foerster B., Richtering W. (2023). Superchaotropic
Nano-ion Binding as a Gelation Motif in Cellulose Ether Solutions. Angew. Chem., Int. Ed..

[23] Bernier N. A., Teh J., Reichel D., Zahorsky-Reeves J. L., Perez J. M., Spokoyny A. M. (2021). Ex Vivo
and In Vivo Evaluation of Dodecaborate-Based Clusters Encapsulated
in Ferumoxytol Nanoparticles. Langmuir.

[24] Dordovic V., Uchman M., Prochazka K., Zhigunov A., Plestil J., Nykanen A., Ruokolainen J., Matejicek P. (2013). Hybrid Nanospheres
Formed by Intermixed Double-Hydrophilic Block Copolymer Poly­(ethylene
oxide)-block-poly­(2-ethyloxazoline) with High Content of Metallacarboranes. Macromolecules.

[25] Kawasaki R., Sasaki Y., Akiyoshi K. (2017). Intracellular
delivery and passive
tumor targeting of a self-assembled nanogel containing carborane clusters
for boron neutron capture therapy. Biochem.
Biophys. Res. Commun..

[26] Hu K., Yang Z., Zhang L., Xie L., Wang L., Xu H., Josephson L., Liang S. H., Zhang M. R. (2020). Boron agents for
neutron capture therapy. Coord. Chem. Rev..

[27] Thomas J., Hawthorne M. F. (2001). Dodeca­(carboranyl)-substituted
closomers: toward unimolecular
nanoparticles as delivery vehicles for BNCT. Chem. Commun..

[28] van
Rossum B. J., Foerster H., de Groot H. J. M. (1997). High-field and
high-speed CP-MAS C-13 NMR heteronuclear dipolar-correlation spectroscopy
of solids with frequency-switched Lee-Goldburg homonuclear decoupling. J. Magnetic Resonance.

[29] Farmer M. A. H., Musa O. M., Armes S. P. (2023). Efficient
Synthesis of Hydrolytically
Degradable Block Copolymer Nanoparticles via Reverse Sequence Polymerization-Induced
Self-Assembly in Aqueous Media. Angew. Chem.,
Int. Ed..

[30] Assaf K. I., Ural M. S., Pan F., Georgiev T., Simova S., Rissanen K., Gabel D., Nau W. M. (2015). Water Structure
Recovery in Chaotropic Anion Recognition: High-Affinity Binding of
Dodecaborate Clusters to g-Cyclodextrin. Angew.
Chem. Int. Ed.

[31] Fanova A., Davidovich I., Talmon Y., Skandalis A., Pispas S., Stepanek M. (2021). Modification of the Co-assembly Behavior
of Double-Hydrophilic Block Polyelectrolytes by Hydrophobic Terminal
Groups: Ordered Nanostructures with Interpolyelectrolyte Complex Domains. ACS Appl. Polym. Mater..

[32] Norisuye T., Inoue M., Shibayama M., Tamaki R., Chujo Y. (2000). Time-Resolved
Dynamic Light Scattering Study on the Dynamics of Silica Gels during
Gelation Process. Macromolecules.

[33] Djabourov M. (1991). Gelation–a
review. Polym. Int..

[34] Brus J., Zhigunov A., Czernek J., Kobera L., Uchman M., Matejicek P. (2014). Control over
the Self-Assembly and Dynamics of Metallacarborane
Nanorotors by the Nature of the Polymer Matrix: A Solid-State NMR
Study. Macromolecules.

[35] Aghaei
Hakkak R., Schleid T. (2024). Crystal structure and thermal behavior
of three potential high-energy compounds of hydro-closo-borates with
guanidinium. J. Solid State Chem..

[36] Song J., Zhang Q., de Quesada F., Rizvi M. H., Tracy J. B., Ilavsky J., Narayanan S., Del Gado E., Leheny R. L., Holten-Andersen N., McKinley G. H. (2022). Microscopic dynamics underlying the
stress relaxation of arrested soft materials. Proc. Natl. Acad. Sci. U.S.A..

[37] Hesarinejad M. A., Koocheki A., Razavi S. M. A. (2014). Dynamic rheological properties of
Lepidium perfoliatum seed gum: Effect of concentration, temperature
and heating/cooling rate. Food Hydrocoll.

[38] Wu Y., Guo R., Cao N., Sun X., Sui Z., Guo Q. (2018). A systematical
rheological study of polysaccharide from Sophora alopecuroides L.
seeds. Carbohydr. Polym..

[39] Khondkar D., Tester R. F., Hudson N., Karkalas J., Morrow J. (2007). Rheological
behaviour of uncross-linked and cross-linked gelatinized waxy maize
starch with pectin gels. Food Hydrocoll.

