# Supplementary material for: Boron-Rich Soft Hydrogels Based on the Coassembly of Cationic A‑B‑A Triblock Copolymers with Closo-Dodecaborate
Source: Macromolecules. 2025 Jul 10;58(14):7276–86. doi: 10.1021/acs.macromol.5c01181 (PMC12288075; doi:10.1021/acs.macromol.5c01181)
Supplement: Supplementary file 1 [file ma5c01181_si_001.pdf]

Supporting Information

Boron-Rich Soft Hydrogels Based on the Coassembly of Cationic A-B-A Triblock Copolymers with *Closo*-Dodecaborate

Soňa Mesíková,[1] Jianwei Li,#[1] Sami Kereïche,[2] Zdeněk Tošner,[3] Mariusz Uchman,[1] Miroslav Štěpánek,[1] Michael Gradzielski,[4] Pavel Matějíček*[1]

[1]*Department of Physical and Macromolecular Chemistry, Faculty of Science, Charles University, Hlavova 2030/8, 128 40 Prague 2, Czechia;* [2]*Institute of Biology and Medical Genetics, First Faculty of Medicine, Charles University and General University Hospital in Prague, Purkynie Ustav, Albertov 4, 12 801 Prague, Czechia;* [3]*NMR Laboratory, Faculty of Science, Charles University, Hlavova 2030/8, 128 40 Prague 2, Czechia;* [4] Stranski-Laboratorium für Physikalische Chemie und Theoretische Chemie, Institut für Chemie, Sekr. TC 7, Technische Universität Berlin, Strasse des 17. Juni 124, D-10623 Berlin, Germany; #Current address: State Key Laboratory of Fine Chemicals, Dalian University of Technology, 2 Linggong Road, Dalian 116024, China.

*email: *pavel.matejicek@natur.cuni.cz*

1 Synthesis and characterization.

1.1 Synthesis. NMR spectra.

1.2 SEC traces.

1.3 Gel-like samples.

2 Isothermal Titration Calorimetry.

2.1 ITC titrations.

3 Dynamic Light Scattering.

3.1 Diffusive character of the modes.

3.2 Results of fitting.
4 SAXS modelling.
4.1 Results of fitting.
5 Structural analysis.
5.1 Ss-NMR spectroscopy.
6 Mechanical properties.
6.1 Rheology experiments in water mixtures.
6.2 Rheology experiments in 0.1 M NaCl solution mixtures.

1 Synthesis and characterization.

1.1 NMR spectra.

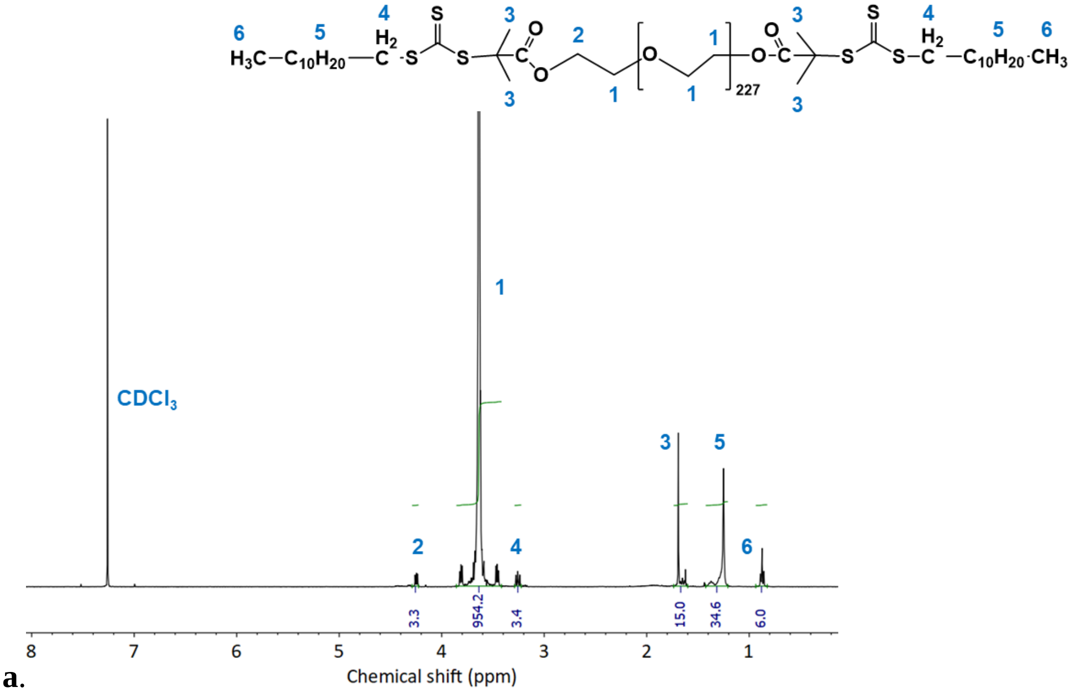

a.

Figure S1a-g. (**a**) ^{1}H NMR spectrum of PEO$_{227}$-(DDMAT)$_2$.

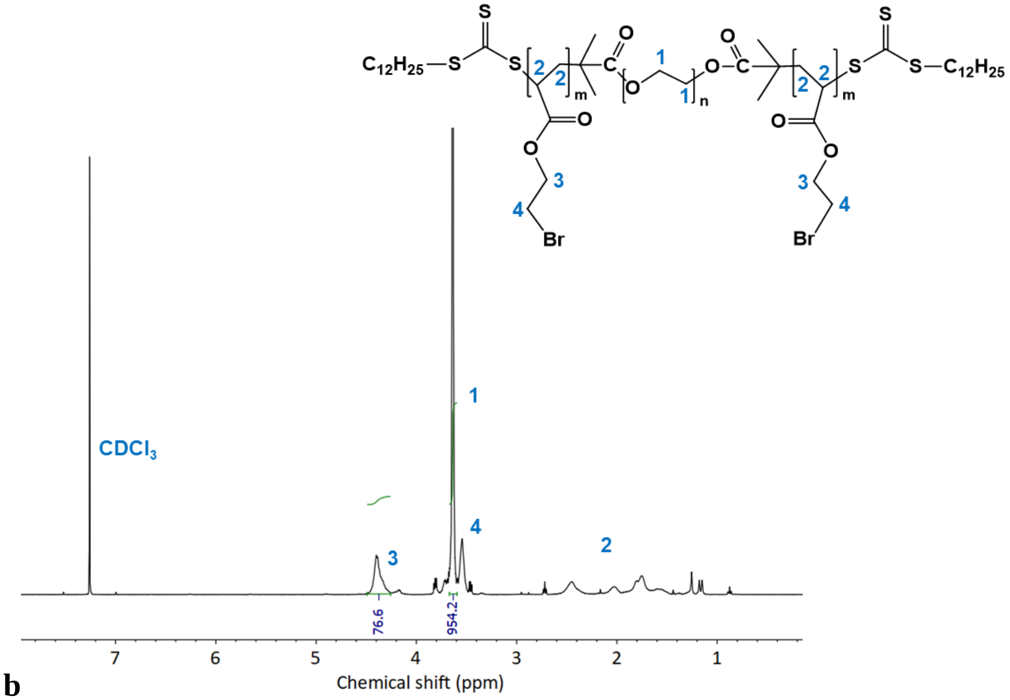

b

Figure S1a-g. (**b**) ^{1}H NMR spectrum of PEO$_{227}$-(PBEA$_{20}$)$_2$.

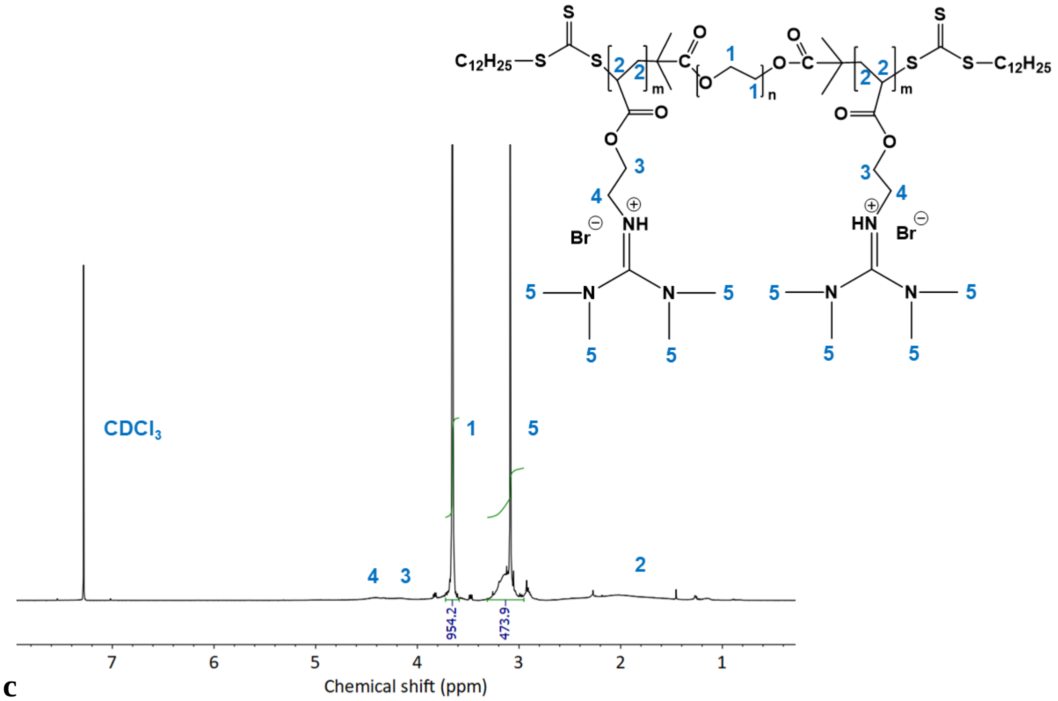

c

Figure S1a-g. (**c**) ^{1}H NMR spectrum of PEO_{227}-$(PGEA_{20})_2$.

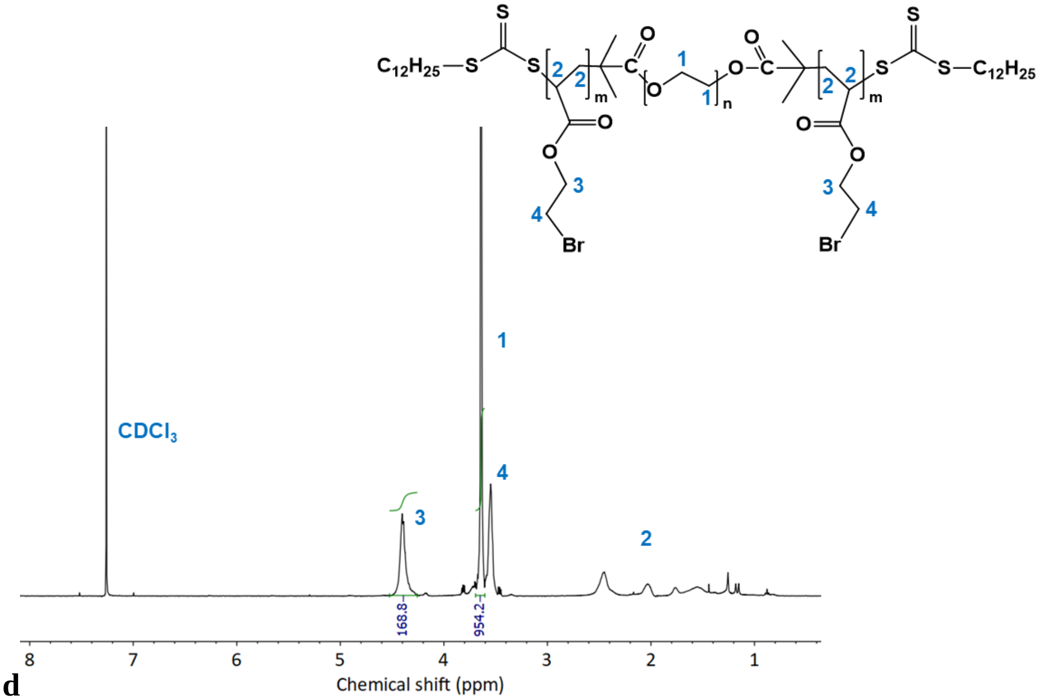

d

Figure S1a-g. (**d**) ^{1}H NMR spectrum of PEO_{227}-$(PBEA_{40})_2$.

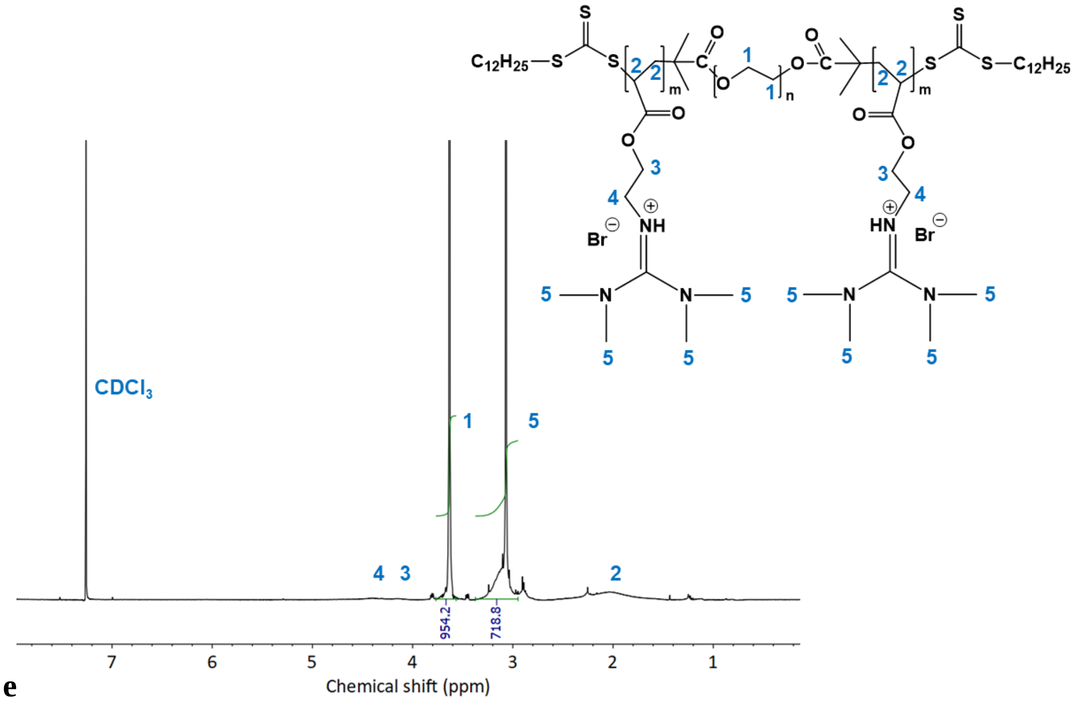

e

Figure S1a-g. (**e**) ^{1}H NMR spectrum of PEO$_{227}$-(PGEA$_{40}$)$_2$.

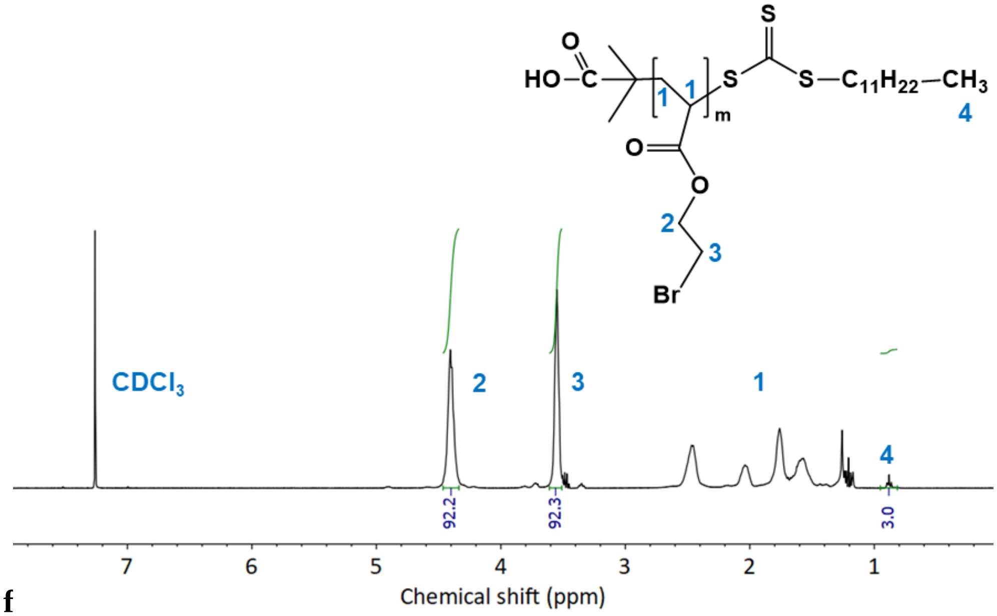

f

Figure S1a-g. (**f**) ^{1}H NMR spectrum of PBEA$_{40}$.

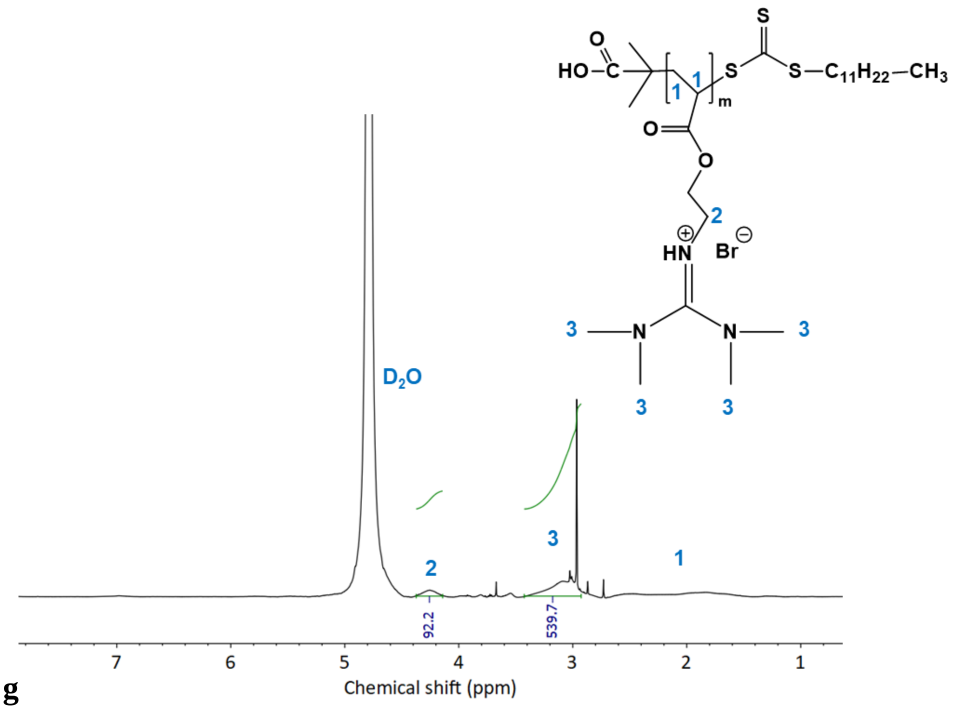

g

Figure S1a-g. **(g)** ^{1}H NMR spectrum of $PGEA_{40}$.

1.2 SEC traces.

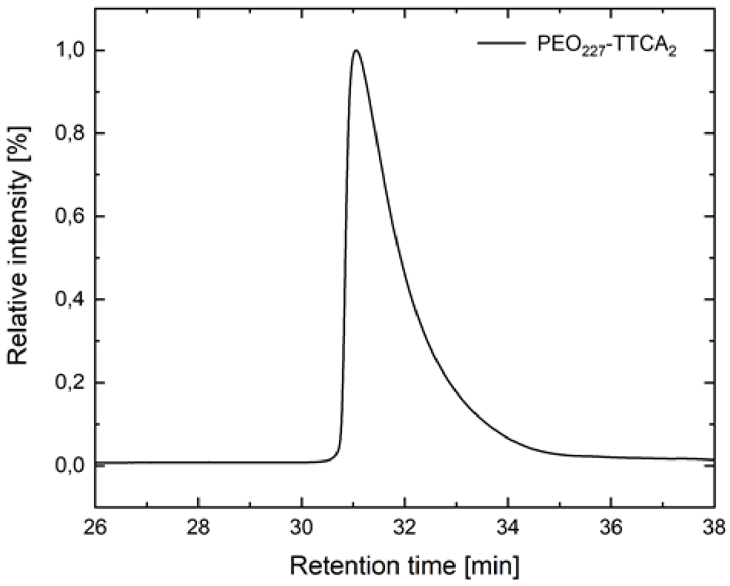

a

Figure S2a-d. (**a**) SEC chromatogram of PEO_{227}-$(DDMAT)_2$; M_n: 7604, M_w: 8489, *Đ* 1.11.

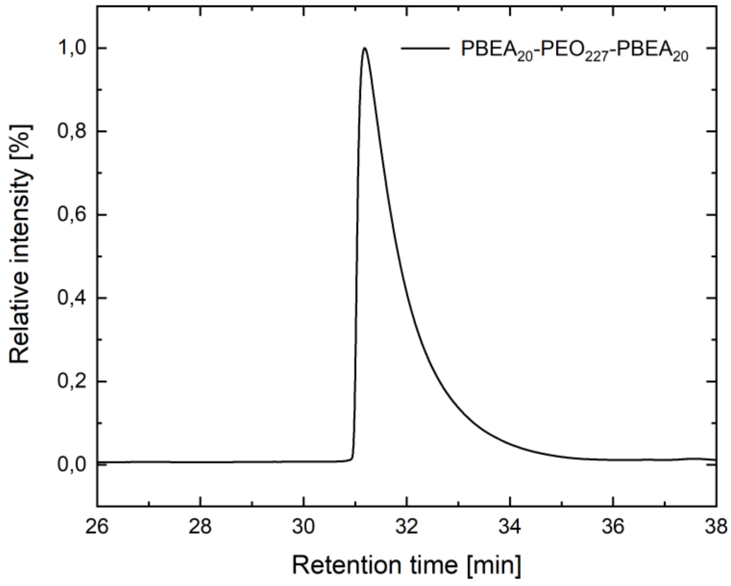

b

Figure S2a-d. (**b**) SEC chromatogram of PEO_{227}-$(PBEA_{20})_2$; M_n: 7735, M_w: 8832, *Đ* 1.14.

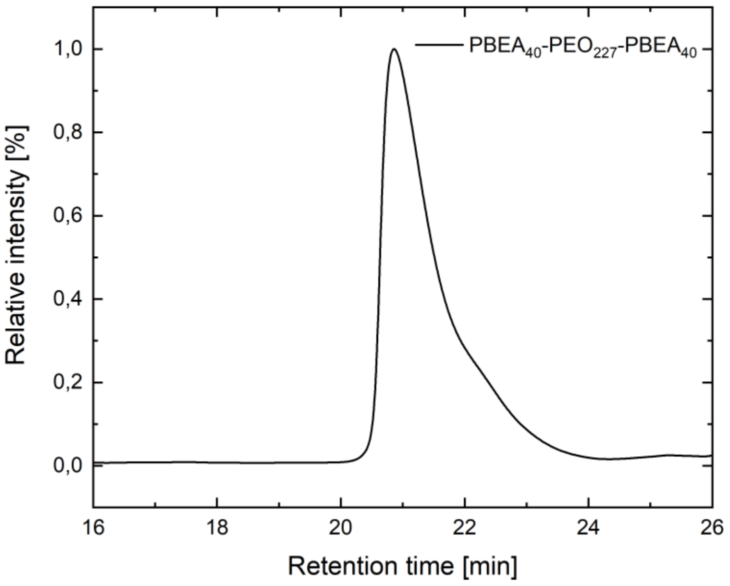

c

Figure S2a-d. (**c**) SEC chromatogram of PEO_{227}-$(PBEA_{40})_2$; M_n: 14433, M_w: 16737, *Đ* 1.16.

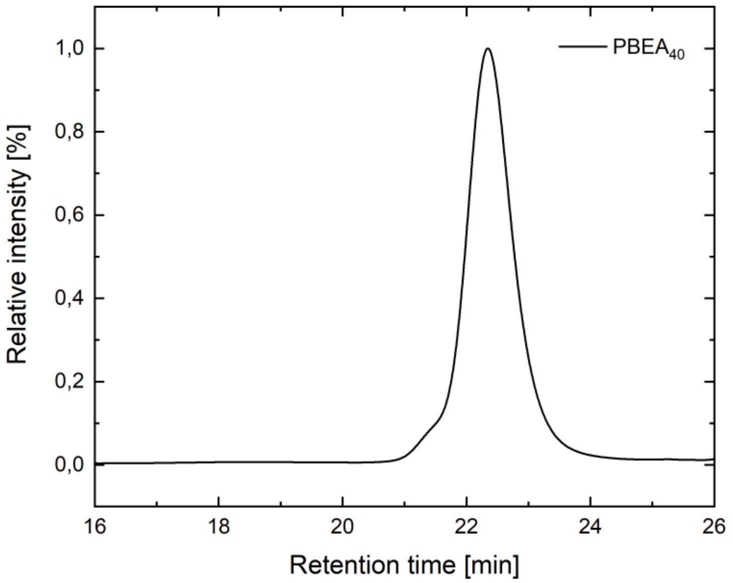

d

Figure S2a-d. (**d**) SEC chromatogram of $PBEA_{40}$; M_n: 7837, M_w: 8480, *Đ* 1.08.

1.3 Gel-like samples.

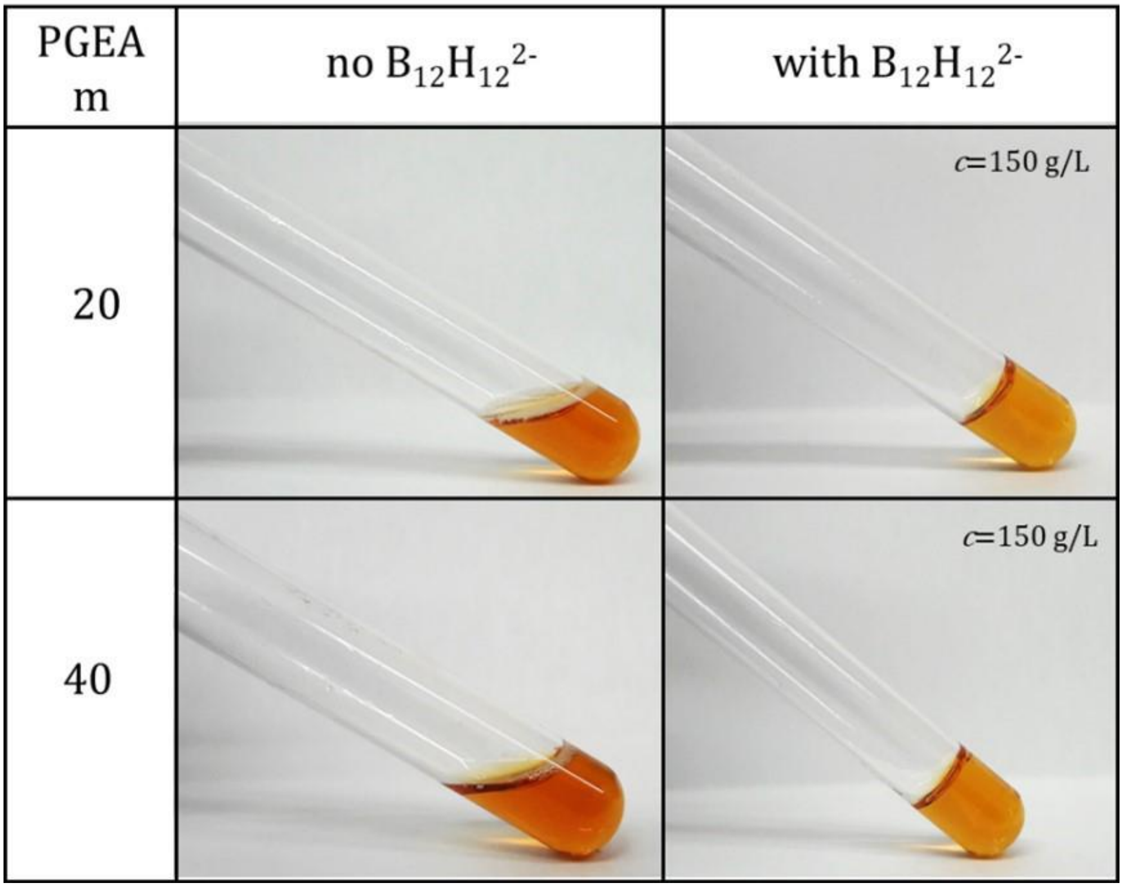

Figure S3. Photographs of the samples in the glass tubes used for DLS measurements. On the left side are samples with polymer concentration 150 g/L without *closo*-dodecaborate. On the right side are the same samples after the addition of disodium *closo*-dodecaborate aqueous solution.

2 Isothermal Titration Calorimetry.

2.1 ITC titrations.

Table S1. Results of fitting of the enthalpograms by one-site binding model providing the dodecaborate-guanidine stoichiometry, n, the heat of nanoparticle formation, ΔH, the association constant, K_a, and related free energy of the association, ΔG, and then the entropy contribution to the free energy of association, $-T\Delta S$.

	Water				
	n	K_a	ΔG kJ/mol	ΔH kJ/mol	$-T\Delta S$ kJ/mol
PGEA20-PEO45-PGEA20	0.11	5×10^4	-26	-23	-3
PGEA20-PEO136-PGEA20	0.07	2×10^4	-25	-36	+11
PGEA20-PEO227-PGEA20	0.04	1×10^4	-24	-45	+21

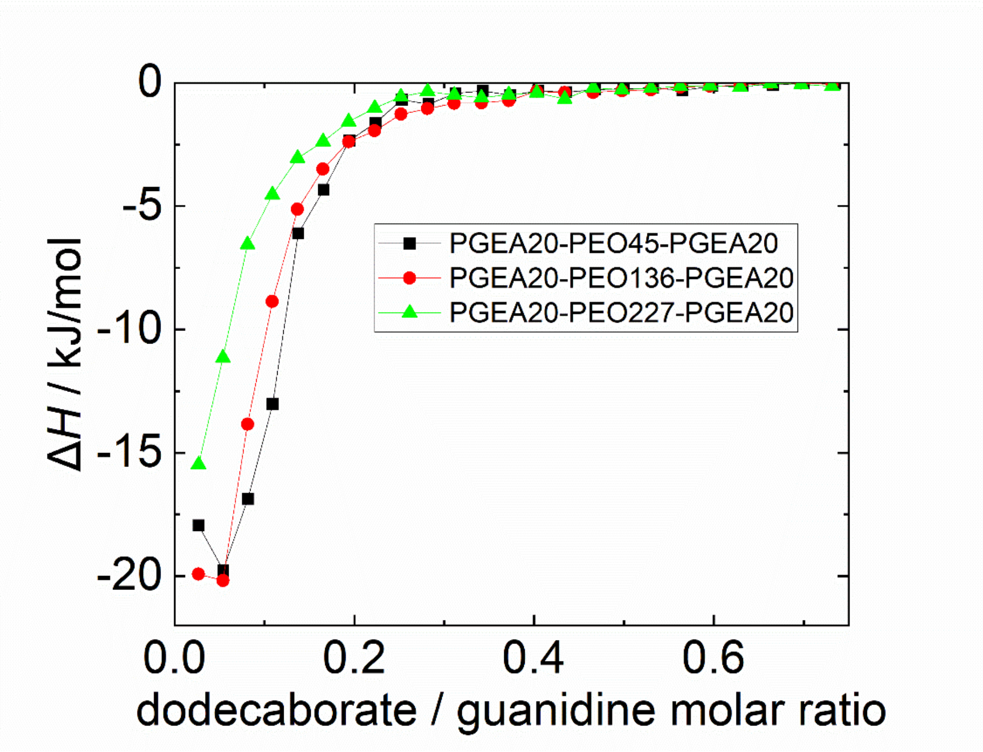

Figure S4. Enthalpograms evaluated from ITC data for the titration of $PGEA_x$-b-PEO_y-b-$PGEA_x$ triblock copolymers as indicated within the graph by disodium *closo*-dodecaborate in pure water solutions.

3 Dynamic Light Scattering.

3.1 Diffusive character of the modes.

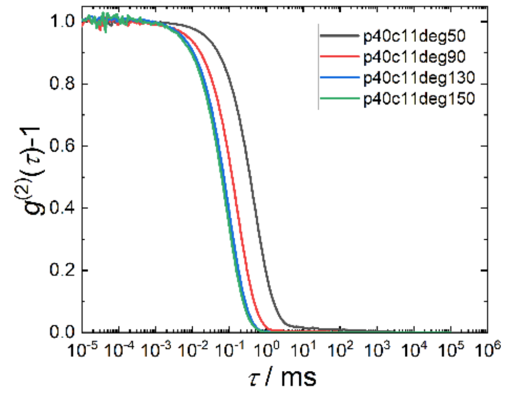

a

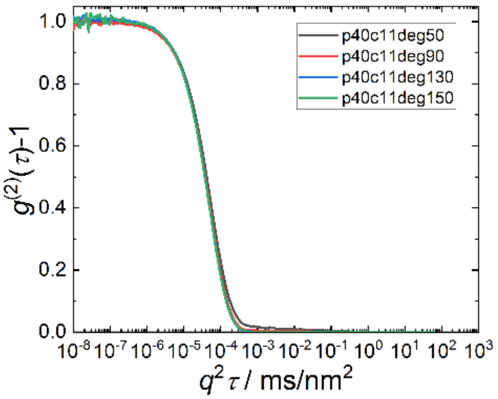

b

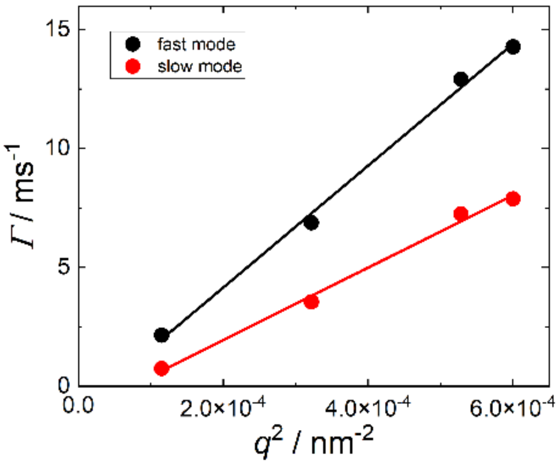

c

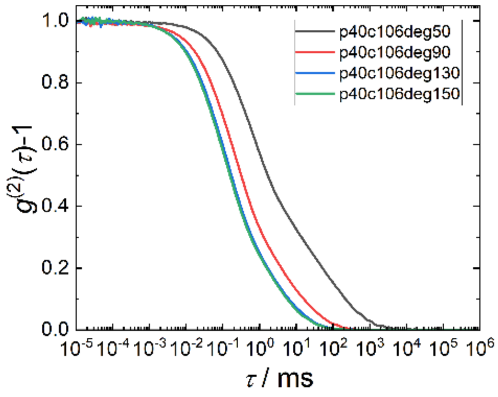

d

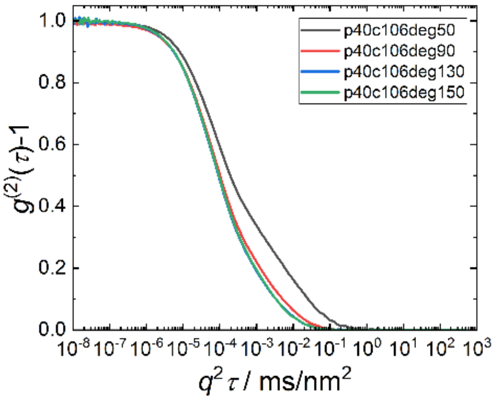

e

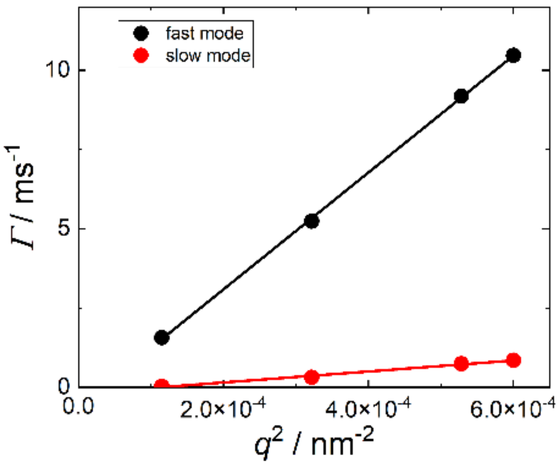

f

Figure S5a-f. Typical (**a,b,d,e**) normalized intensity autocorrelation functions $g^{(2)}$ and (**c,f**) the q^2-dependence of the correlation rates Γ for PGEA40-PEO-PGEA40/B12 samples at polymer concentrations (**a-c**) 11 g/L and (**d-f**) 106 g/L.

3.2 Results of fitting.

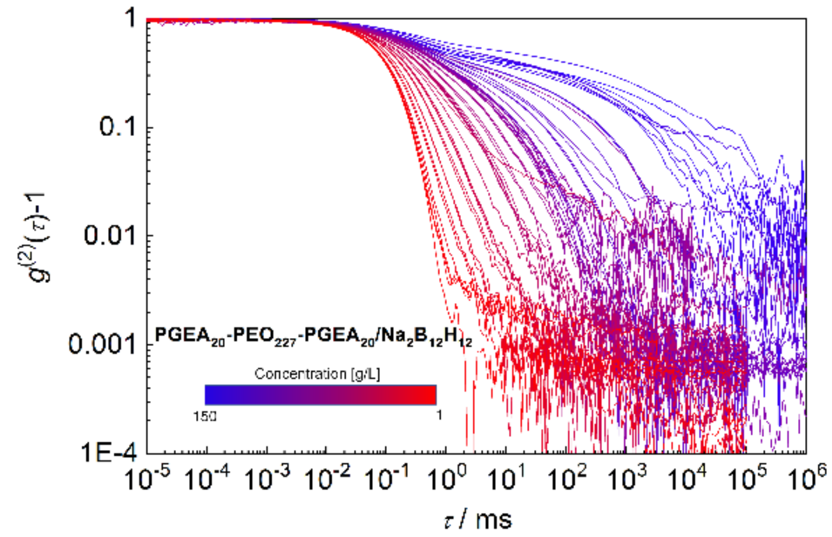

a

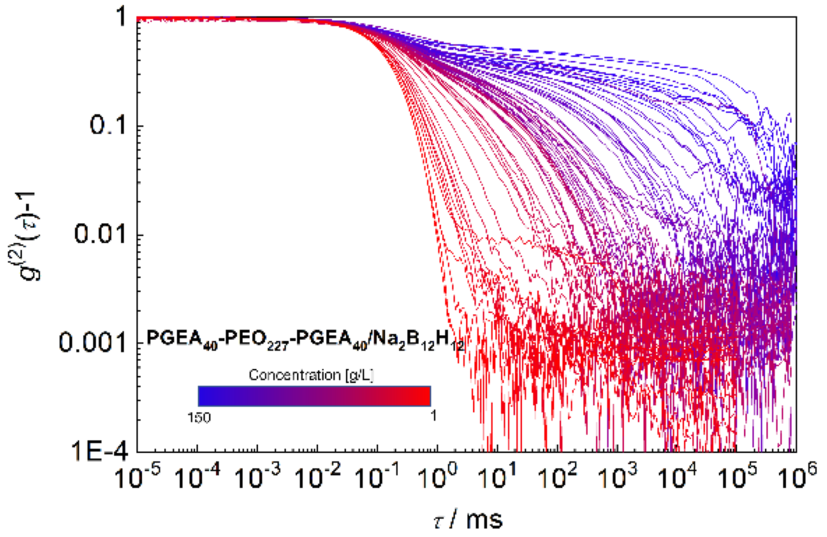

b

Figure S6a,b. Normalized intensity autocorrelation functions in the LOG-LOG representation of systems (**a**) $PGEA_{20}$-PEO-$PGEA_{20}$/B12 and (**b**) $PGEA_{40}$-PEO-$PGEA_{40}$/B12 at the range of concentrations from 150 to 1 g/L.

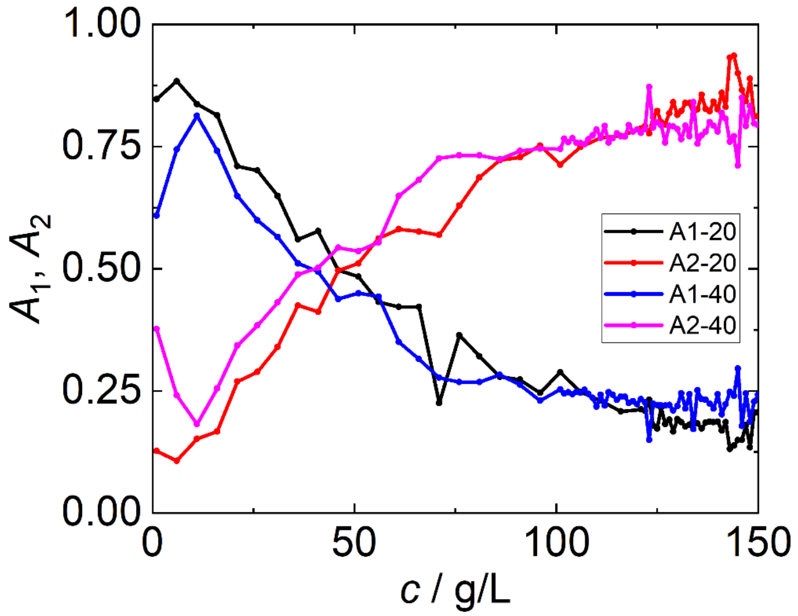
1.00
0.75
0.50
0.25
0.00
0
50
100
150
A_1, A_2
c / g/L
A1-20
A2-20
A1-40
A2-40

a

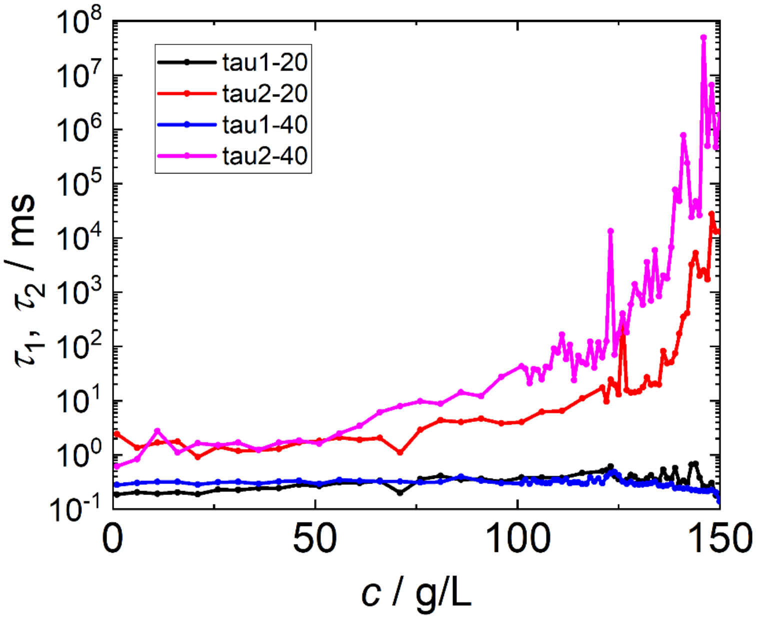
10^8
10^7
10^6
10^5
10^4
10^3
10^2
10^1
10^0
10^{-1}
0
50
100
150
τ_1, τ_2 / ms
c / g/L
tau1-20
tau2-20
tau1-40
tau2-40

b

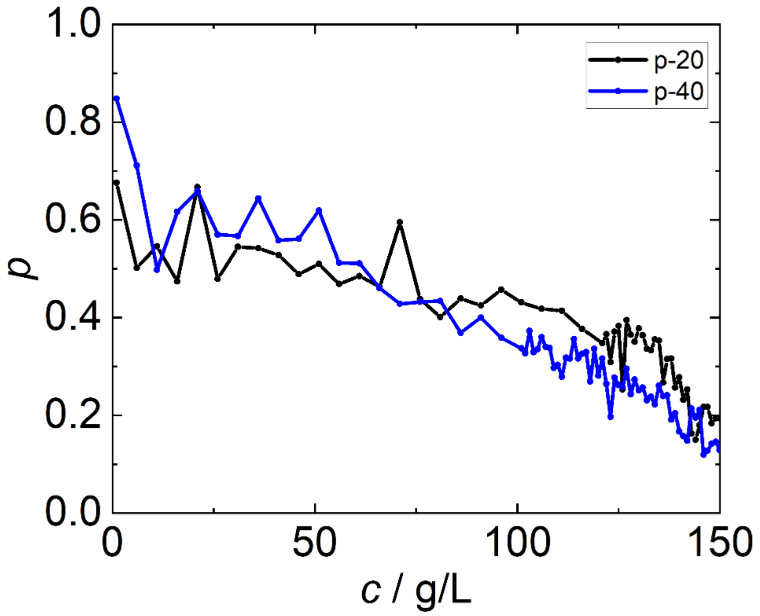

c

Figure S7a-c. The results of fitting of normalized intensity autocorrelation functions (Figures 1 and S6) by a combination of exponential and stretched exponential function as follows: $[g^{(2)}-1]^{1/2} = A_1\exp[-t/\tau_1]+A_2\exp[-(t/\tau_2)^p]$, where (**a**) A_1 and A_2 are the amplitudes of the fast and slow modes, respectively; (**b**) τ_1 and τ_2 are the correlation times of the fast and slow modes, respectively; and (**c**) p is the stretched exponent of the slow mode.

4 SAXS modelling.

4.1 Results of fitting.

Table S2. Results of fitting of SAXS curves shown in Figures 3a,b by the model for dilute liquid samples described in Experimental section.

**Sample (pure water)**		R_{core} (nm)	ΔR_{shell} (nm)	$l_{cylinder}$ (nm)	R_{HS} (nm)	φ	χ^2
	c **(g/L)**	**mean**	**mean**	**mean**	**mean**	**mean**	
PGEA20-PEO227-PGEA20/B12	10	2.669	3.973	19.25			1.54
	40	2.531	3.786	13.88	10.79	0.0621	1.78
	80	2.175	3.491	11.54	9.05	0.097	2.65
PGEA40-PEO227-PGEA40/B12	10	2.372	4.156	39.61			3.14
	40	2.619	4.117	38.71			6.69
	80	3.06	4.321	11.41			4.83

Table S3. Results of fitting of SAXS curves shown in Figures 3c by the model for gel-like samples described in Experimental section.

**Sample (gel)**	***c*** **(g/L)**	I_{max} (a.u.)	ξ (nm)	q_{max} (nm^{-1})	m	p	I_b (a.u.)	I_1 (a.u.)	α
PGEA20-PEO227-PGEA20/B12	150	1.665	8.7	0.332	1.61	1.52	0.0197	0.000233	4.13
PGEA40-PEO227-PGEA40/B12	150	4.64	11	0.268	2.05	1.17	0.0679	0.0238	2.42

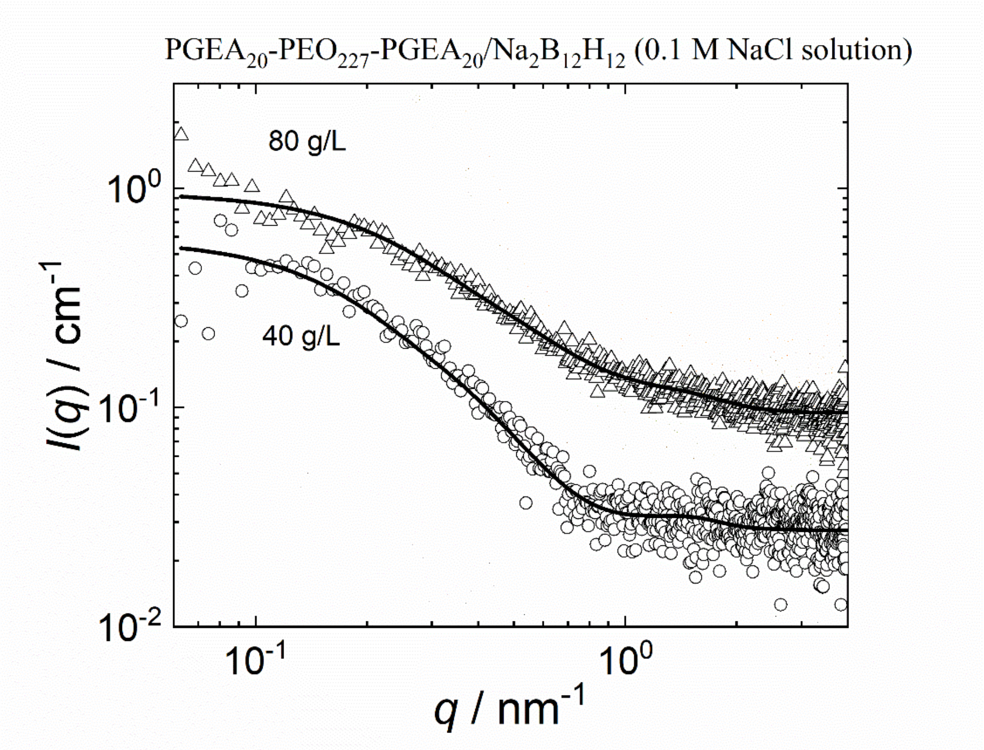

a

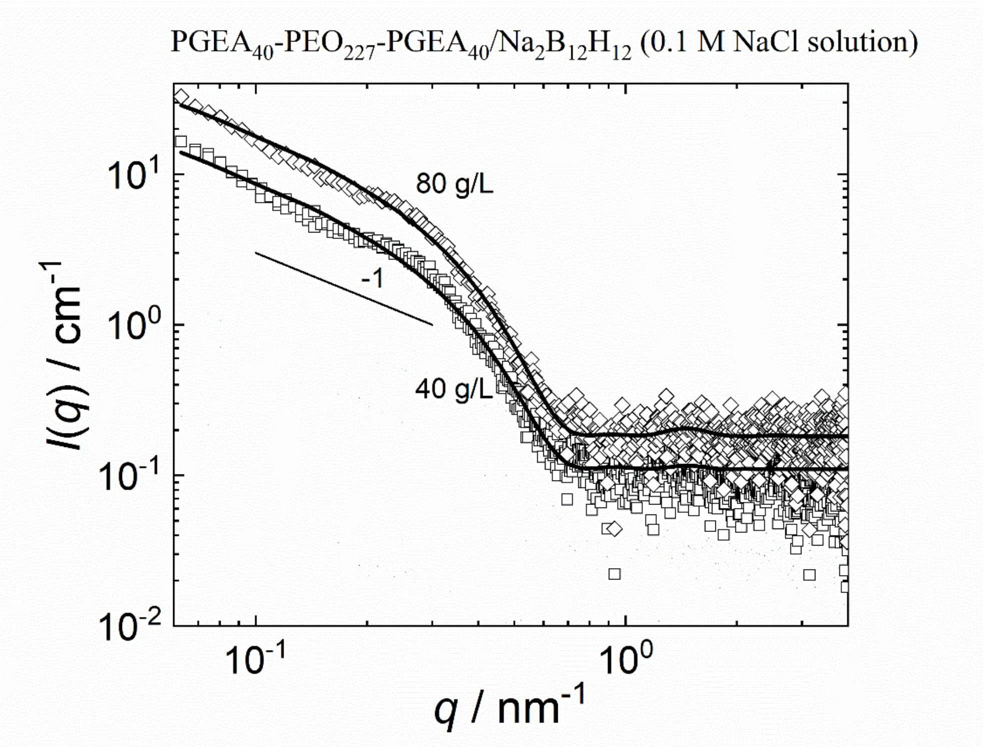

b

Figure S8a,b. SAXS curves with corresponding fits of liquid samples of (**a**) $PGEA_{20}$-PEO-$PGEA_{20}$/B12 and (**b**) $PGEA_{40}$-PEO-$PGEA_{40}$/B12 in the concentration range 10-80 g/L in 0.1 M NaCl solution.

Table S4. Results of fitting of SAXS curves shown in Figures S8a,b by the model for dilute liquid samples described in Experimental section.

Sample (0.1 M NaCl)		R_{core} (nm)	ΔR_{shell} (nm)	$l_{cylinder}$ (nm)	χ^2
	c (g/L)	mean	mean	mean	
PGEA20-PEO227-PGEA20/B12	40	1.102	4.04	17.12	1.69
	80	1.095	3.82	17.12	1.48
PGEA40-PEO227-PGEA40/B12	40	1.684	3.85	68.5	3.32
	80	0.971	4.61	66.3	2.16

The electrostatic character of PGEA/B12 complexation was tested by SAXS scattering experiments with the PGEA-PEO-PGEA/B12 nanoparticles in 0.1 M NaCl solutions (Figures S8 and evaluation in Table S4). In general, the absolute signal intensity is weaker in comparison to samples in pure water. Thus, the data for the lowest polymer concentration (10 g/L) were not evaluated. It was possible to fit the scattering curves by the model of core/shell cylinders, nevertheless the particles have thinner cores and their length is rather nonuniform in comparison to the system in pure water.

5 Structural analysis.

5.1 ss-NMR spectroscopy.

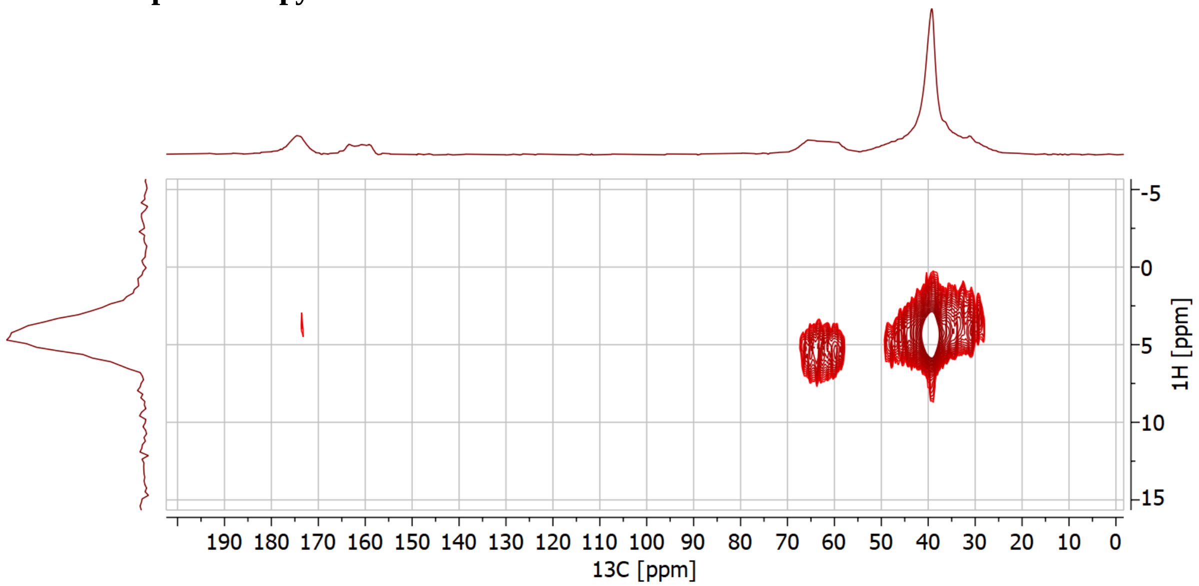

Figure S9. 2D ^{1}H-^{13}C HETCOR spectrum of PGEA$_{40}$/B12. The top spectrum is a result of ^{1}H-^{13}C CP MAS experiment measured separately. The vertical trace corresponds to projection from the 2D data. The strong resonance at 4.4 ppm / 39 ppm (^{1}H / ^{13}C) is assigned to guanidinium methyl groups. The CH and CH_2 groups of the polymer resonate at around 5.3 ppm / 63 ppm (^{1}H / ^{13}C). The carboxyl and the guanidinium carbons have resonances at 175 ppm and 163 ppm respectively, and do not show any correlations due to a larger distance to nearby protons.

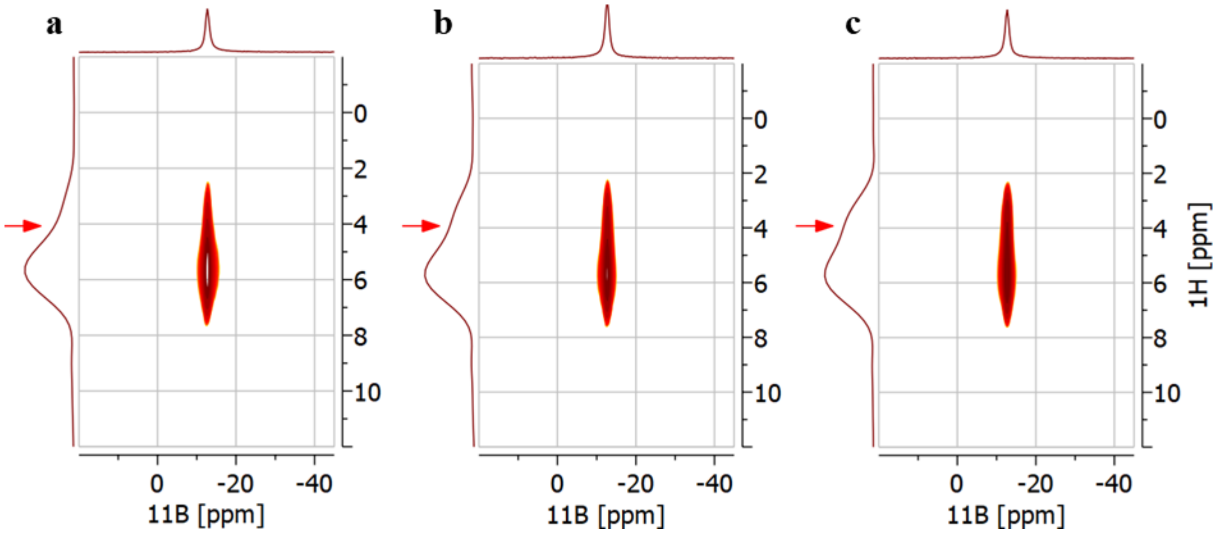

Figure S10a-c. 2D ^{1}H-^{11}B HETCOR spectra of PGEA$_{40}$/B12 with proton spin diffusion element. The period for spin diffusion increases from left to right and is (**a**) 150 μs, (**b**) 500 μs, and (**c**) 2000 μs. Vertical traces are taken at the cross-peak maximum, and the red arrow indicates the growing resonance at about 4 ppm which is corresponding to methyl groups of the guanidinium moiety. This observation indicates their spatial proximity.

6 Mechanical properties.

6.1 Rheology experiments in water mixtures.

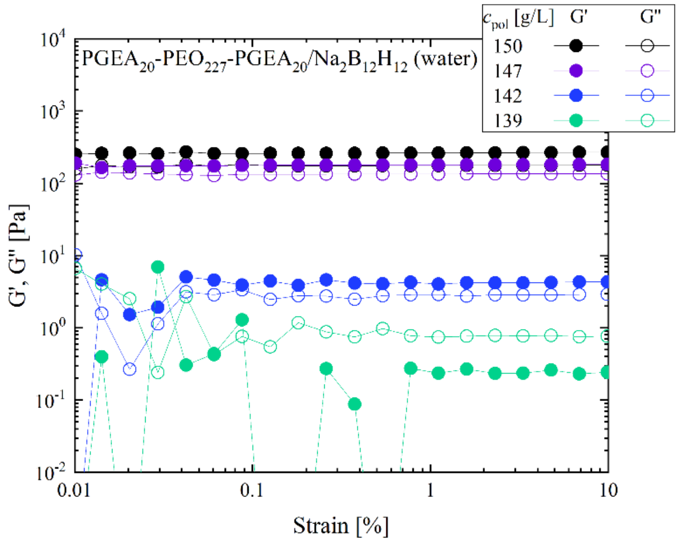

a

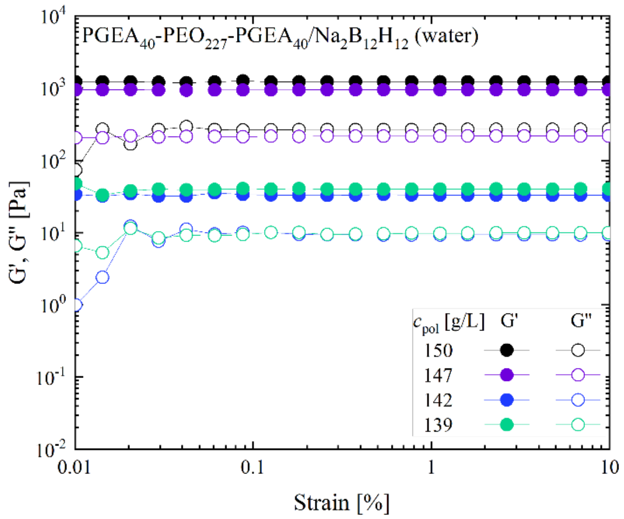

b

Figure S11a,b. Amplitude sweep for (**a**) PGEA20 and (**b**) PGEA40 samples at concentrations 150, 147, 142, 139 and 135 g/L in pure water, where G' is storage modulus and G'' is loss modulus (T = 25 °C).

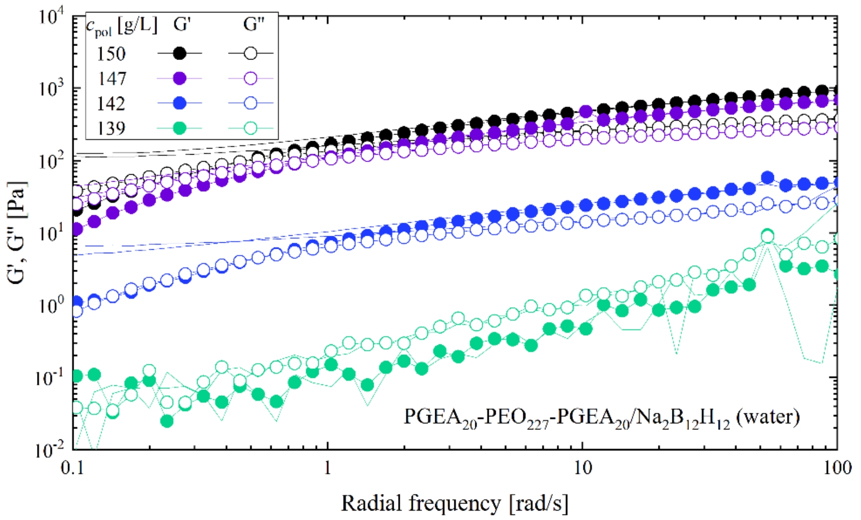

a

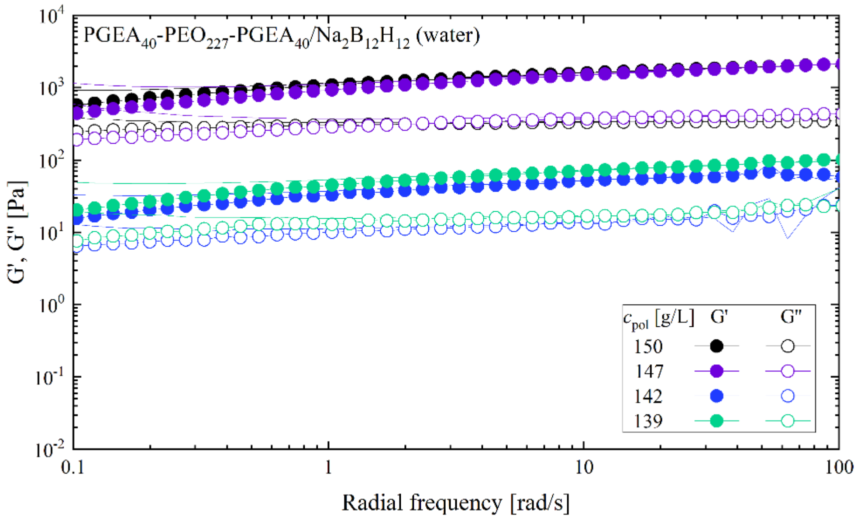

b

Figure S12a,b. Frequency sweep for (**a**) PGEA20 and (**b**) PGEA40 samples at concentrations 150, 147, 142 and 139 g/L in pure water, where *G*' is storage modulus and *G*" is loss modulus. Experiments were done with a deformation of 1% and at T = 25 °C.

G′ curves were fitted by function $G'=A+B\omega^C$, the values of power-law parameter *C* and standard deviation (SD) are listed in **Table 1** below.

Where for physical gels *C*>0. Parameter *C* also determine the strength of the gel, low values of *C* are assigned to elastic gels while viscous gels are close to 1.

PGEA40 (in water) forms elastic gels at all the measured concentrations, PGEA20 also forms elastic gels at high polymer concentrations which transition to viscous gel (139 g/L). Similarly, for

the samples in salt, but transition to viscous gel is observed at higher polymer concentration compared to samples in water. For PGEA40 in salt at concentration 139 g/L parameter *C* is around 2, and better description would be dilute solution.

(For concentration 139 g/L some fits are missing, due to the datapoint fluctuations.)

Table S5: Parameter *C* from frequency sweep measurements fitted by function $G'=A+B\omega^{C}$.

Sample	*c* [g/L]	*C*	SD
PGEA20 (in water)	150	0.201	0.003
	147	0.19	0.03
	142	0.31	0.05
	139	1.0	0.3
PGEA40 (in water)	150	0.00132	0.00128
	147	0.046	0.002
	142	0.041	0.009
	139	0.043	0.009

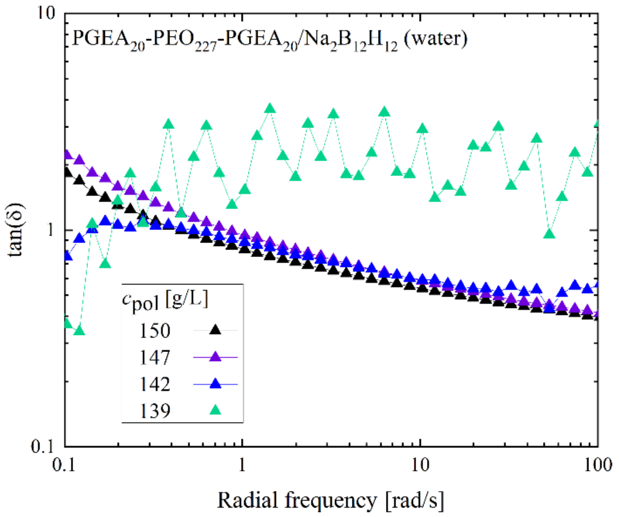

a

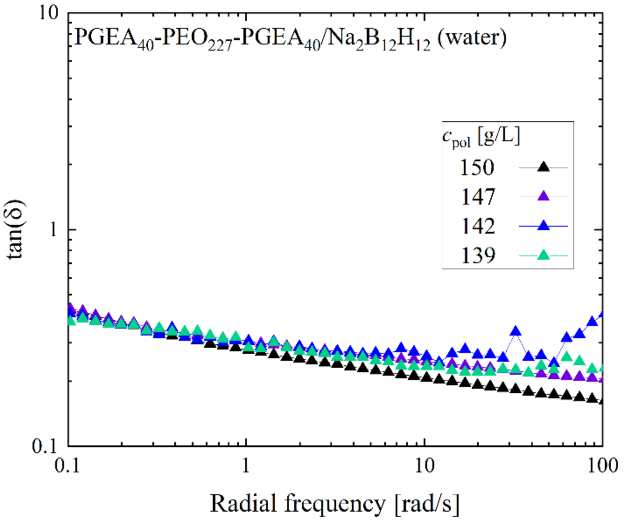

b

Figure S13a,b. Tan(δ) for (**a**) PGEA20 and (**b**) PGEA40 samples at concentrations 150, 147 and 142 g/L in pure water (deformation = 1%, T = 25 °C).

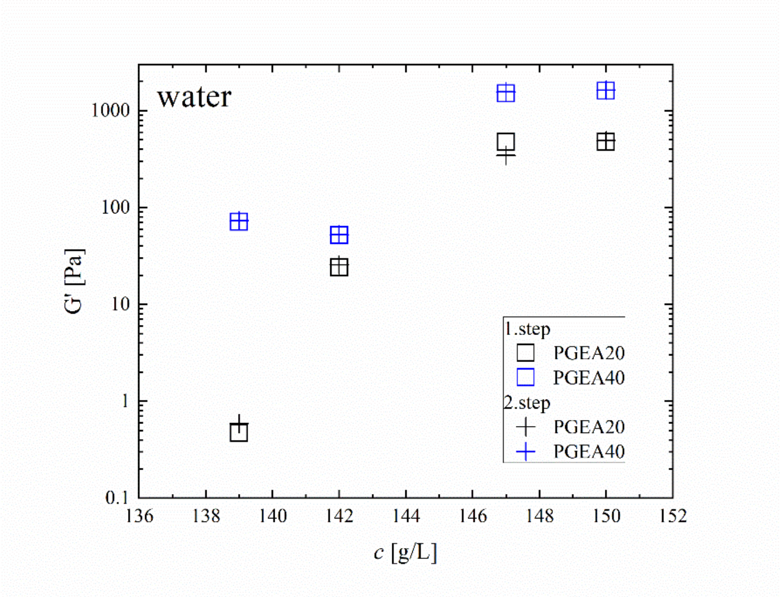

Figure S14. Storage modulus *G'* for the PGEA20 and PGEA40 samples in pure water for different concentrations at *radial* frequency 10 *rad/s and* T = *25* °C; 1. step is frequency sweep from low to high frequencies, 2. step is from high to low frequencies. For samples PGEA20 with concentration 139 g/L in 1. step and for sample PGEA40 with concentration 135 g/L in 2. step, the values of storage modulus are not present due to the big fluctuations at this frequency.

6.2 Rheology experiments in 0.1 M NaCl solution mixtures.

For samples containing 0.1 M NaCl the values of both storage modulus and loss modulus are lower in comparison to samples without added NaCl. For the lowest concentrations in both samples, values of *G'* and *G"* are fluctuating, mainly the values of *G'*, because of the lower elastic properties of the samples. For both PGEA40 and PGEA20 samples in salt solution with concentrations 150 g/L, and for PGEA40 sample in salt solution for the concentration 147 g/L, values of *G'* were higher than values of *G"*, they are more solid-like. The lower concentrations for both samples show higher *G"* than *G'*, they are more liquid like.

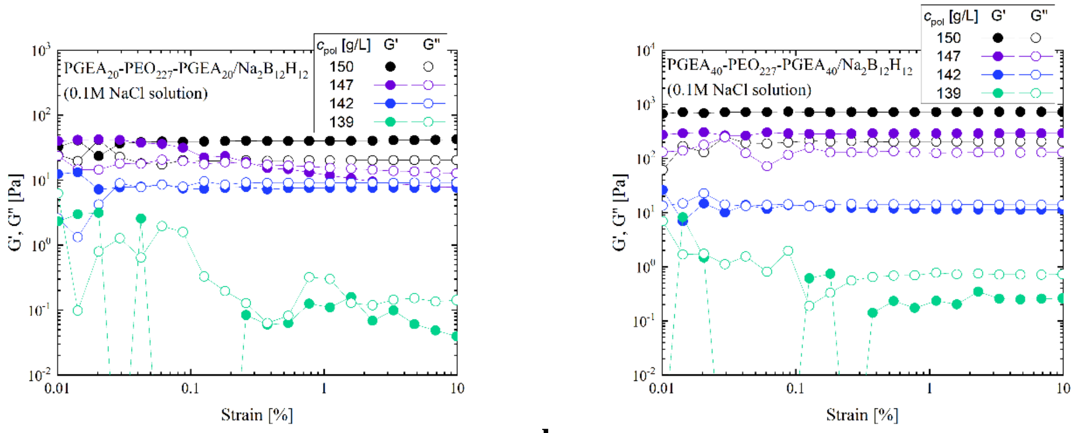

a **b**

Figure S15. Amplitude sweep for (**a**) PGEA20 and (**b**) PGEA40 samples at concentrations 150, 147, 142, 139 and 135 g/L in 0.1 M NaCl solution, where *G'* is storage modulus and *G"* is loss modulus (radial frequency = 10 rad/s, *T* = 25 °C).

For samples in 0.1 M NaCl solution, the values of storage modulus and loss modulus are again lower compared to samples in pure water. We also observe more fluctuations for samples with polymer concentrations 139 and 135 g/L as here with the measurement system employed no reliable measurements could be done. For sample PGEA20 *G'* is higher than *G"* only for concentration 150 g/L. For sample PGEA40, *G'* is higher than *G"* for concentrations 150 and 147 g/L.

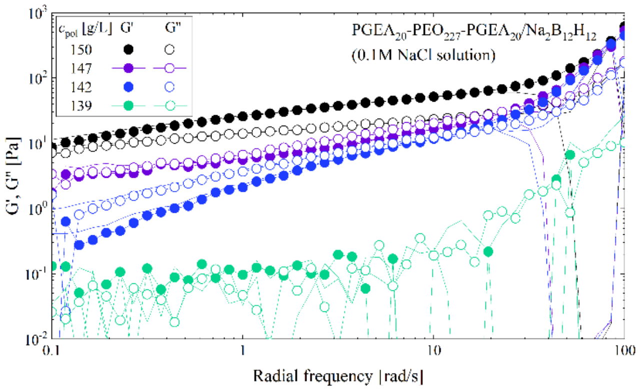

a

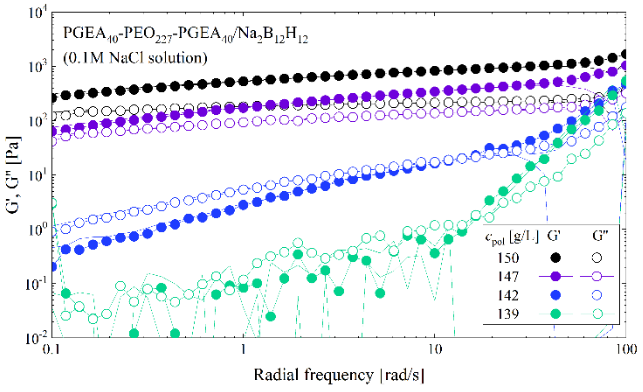

b

Figure S16a,b. Frequency sweep for (**a**) PGEA20 and (**b**) PGEA40 samples at concentrations 150, 147, 142, 139 and 135 g/L in 0.1 M NaCl solution, where *G'* is storage modulus and *G''* is loss modulus. Experiments were done with a deformation of 1% and at *T* = 25 °C.

G' curves were fitted by function $G'=A+B\omega^C$, the value of power-law parameter *C* is listed in **Table 1** below.

Where for physical gels *C*>0. Parameter *C* also determine the strength of the gel, low values of *C* are assigned to elastic gels while viscous gels are close to 1.

PGEA40 (in water) forms elastic gels at all the measured concentrations, PGEA20 also forms elastic gels at high polymer concentrations which transition to viscous gel (139 g/L). Similarly, for the samples in salt, but transition to viscous gel is observed at higher polymer concentration compared to samples in water. For PGEA40 in salt at concentration 139 g/L parameter *C* is around 2, and better description would be dilute solution.

(For concentration 139 g/L some fits are missing, due to the datapoint fluctuations.)

Table S6: Parameter *C* from frequency sweep measurements fitted by function $G'=A+B\omega^C$.

**Sample**	***c* [g/L]**	***C***	**SD**
PGEA20 (in 0.1 M NaCl)	150	0.30	0.02
	147	0.78	0.02
	142	0.68	0.02
	139	-	-
PGEA40 (in 0.1 M NaCl)	150	0.27	0.03
	147	0.52	0.05
	142	0.73	0.03
	139	1.9	0.3

For storage modulus at radial frequency 10 rad/s, we see that the decrease of the storage modulus with decreasing concentration is generally much more pronounced that in the case of pure water and it is again sharper for PGEA20. The length of the PGEA block clearly has an impact on the strength of interactions within the PGEA/dodecaborate domains.

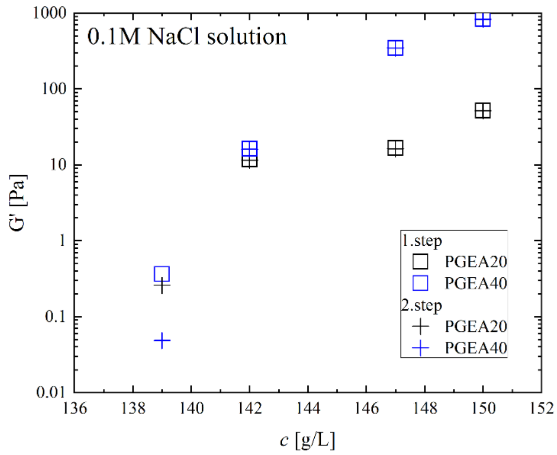

Figure S17. Storage modulus *G'* for PGEA20 and PGEA40 samples in 0.1 M NaCl solution for different concentrations at radial frequency 10 rad/s; 1. step is frequency sweep from low to high frequencies, 2. step is from high to low frequencies (T = 25 °C).

The differences in tan(δ) values for different concentrations are more prominent in salt solution. Sample PGEA20 at concentration 150 g/L has tan(δ) lower than 1 ($G''<G'$), at concentrations 147 and 142 g/L tan(δ) higher than 1 ($G''>G'$), except for higher frequencies. Sample PGEA40 at

concentrations 150 and 147 g/L has tan(δ) lower than 1 ($G''<G'$), at concentration 142 g/L tan(δ) higher than 1 ($G''>G'$), except for higher frequencies. Concentrations 139 and 135 g/L are not shown for either of the samples because of the fluctuation of the data.

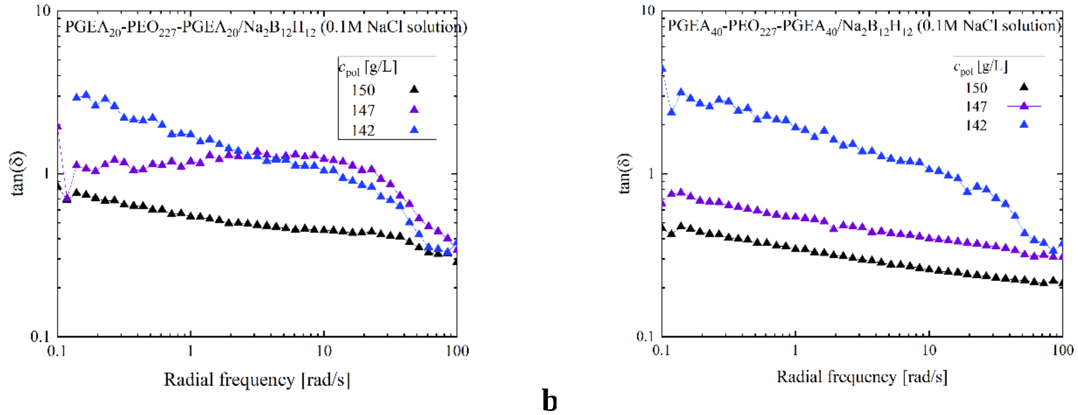

a b

Figure S18a,b. Tan(δ) for (**a**) PGEA20 and (**b**) PGEA40 samples at concentrations 150, 147 and 142 g/L in 0.1 M NaCl solution (T = 25 °C).

For samples in 0.1 M NaCl solution, the viscosities are lower compared to the samples in pure water. For both polymers, viscosity drops by several orders of magnitude for concentrations 150, 147 for sample PGEA40 and concentration 150 for PGEA20. For other sample PGEA20 the viscosity does not change significantly during the measurement.

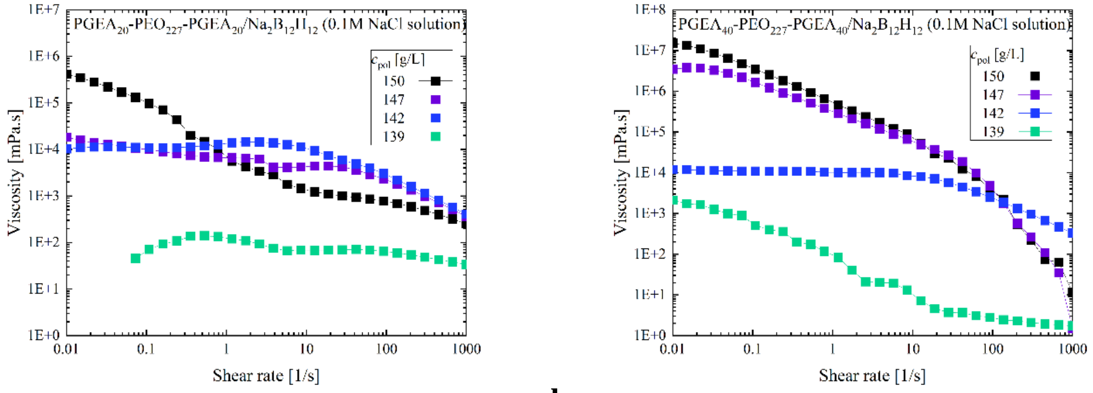

a b

Figure S19a,b. Shear experiment for (**a**) PGEA20 and (**b**) PGEA40 samples at concentrations 150, 147, 142, 139 and 135 g/L in 0.1 M NaCl solution (T = 25 °C).

In 0.1 M NaCl solution, all samples PGEA20 and PGEA40 at concentrations 142, 139 and 135 g/L show almost no increase in values of storage and loss modulus over the course of the 30-minute self-healing test. These samples did not reach high values of storage and loss modulus in frequency sweep and the shear deformation that was put on them prior to self-healing test might not have been enough to break the gel network. Samples with concentrations 150 and 147 g/L show increase of storage and loss moduli but do not reach the values from frequency sweep at 10 rad/s.

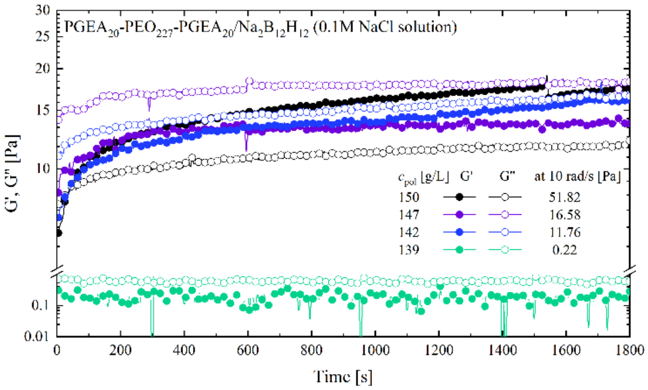

a

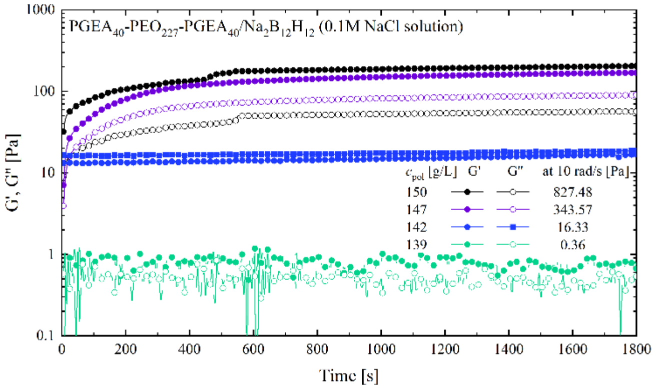

b

Figure S20a,b. Self-healing test for (**a**) PGEA20 and (**b**) PGEA40 samples at concentrations 150, 147, 142, 139 and 135 g/L in 0.1 NaCl solution, where storage modulus is represented by full lines and loss modulus is represented by dotted lines. Full squares represent storage modulus obtained from frequency sweep at radial frequency 10 rad/s. (T = 25 °C).
